# Citrullinated and malondialdehyde-acetaldehyde modified fibrinogen activates macrophages and promotes an aggressive synovial fibroblast phenotype in patients with rheumatoid arthritis

**DOI:** 10.3389/fimmu.2023.1203548

**Published:** 2023-08-16

**Authors:** Nozima Aripova, Michael J. Duryee, Bryant R. England, Carlos D. Hunter, Jack E. Mordeson, Evan M. Ryan, Eric C. Daubach, Debra J. Romberger, Geoffrey M. Thiele, Ted R. Mikuls

**Affiliations:** ^1^ Department of Internal Medicine, Division of Rheumatology, University of Nebraska Medical Center, Omaha, NE, United States; ^2^ Department of Research Services 151, Veteran Affairs Nebraska-Western Iowa Health Care System, Omaha, NE, United States; ^3^ Department of Internal Medicine, Division of Pulmonary, Critical Care, and Sleep Medicine, Omaha, NE, United States

**Keywords:** rheumatoid arthritis, human fibroblast-like synoviocyte, citrullination, malondialdehyde-acetaldehyde, platelet derived growth factor

## Abstract

**Objective:**

Post-translational protein modifications with malondialdehyde-acetaldehyde (MAA) and citrulline (CIT) are implicated in the pathogenesis of rheumatoid arthritis (RA). Although precise mechanisms have not been elucidated, macrophage-fibroblast interactions have been proposed to play a central role in the development and progression of RA. The purpose of our study was to evaluate the downstream effects of macrophage released soluble mediators, following stimulation with fibrinogen (FIB) modified antigens, on human fibroblast-like synoviocytes (HFLS).

**Methods:**

PMA-treated U-937 monocytes (Mϕ) and macrophage-differentiated peripheral blood mononuclear cells (MP) were stimulated with FIB, FIB-MAA, FIB-CIT, or FIB-MAA-CIT. HFLS-RA cells were stimulated directly with FIB antigens or with supernatants (SN) from macrophages (Mϕ-SN or MP-SN) stimulated with FIB antigens. Genes associated with an aggressive HFLS phenotype, extracellular matrix proteins, and activated signaling pathways were evaluated.

**Results:**

HFLS-RA cells treated with Mϕ-SN^FIB-CIT^ and Mϕ-SN^FIB-MAA-CIT^ demonstrated significant increases in mRNA expression of genes associated with an aggressive phenotype at 24-h as compared to direct stimulation with the same antigens. Similar results were obtained using MP-SN. Cellular morphology was altered and protein expression of vimentin (p<0.0001 vs. Mϕ-SN^FIB^) and type II collagen (p<0.0001) were significantly increased in HFLS-RA cells treated with any of the Mϕ-SN generated following stimulation with modified antigens. Phosphorylation of JNK, Erk1/2, and Akt were increased most substantially in HFLS-RA treated with Mϕ-SN^FIB-MAA-CIT^ (p<0.05 vs Mϕ-SN^FIB^). These and other data suggested the presence of PDGF-BB in Mϕ-SN. Mϕ-SN^FIB-MAA-CIT^ contained the highest concentration of PDGF-BB (p<0.0001 vs. Mϕ-SN^FIB^) followed by Mϕ-SN^FIB-CIT^ then Mϕ-SN^FIB-MAA^. HFLS-RA cells treated with PDGF-BB showed similar cellular morphology to the Mϕ-SN generated following stimulation with modified FIB, as well as the increased expression of vimentin, type II collagen, and the phosphorylation of JNK, Erk1/2 and Akt signaling molecules.

**Conclusion:**

Together, these findings support the hypothesis that in response to MAA-modified and/or citrullinated fibrinogen, macrophages release soluble factors including PDGF-BB that induce fibroblast activation and promote an aggressive fibroblast phenotype. These cellular responses were most robust following macrophage activation with dually modified fibrinogen, compared to single modification alone, providing novel insights into the combined role of multiple post-translational protein modifications in the development of RA.

## 1 Introduction

Rheumatoid arthritis (RA) is a systemic, immune-mediated disease primarily characterized by perpetual synovial inflammation that results in polyarticular arthritis, joint erosions and bone loss (1–3). The transition from asymptomatic preclinical disease to clinically apparent RA is often heralded by the appearance of low-grade synovial inflammation that initially evades detection via clinical examination, but that later progresses into highly characteristic inflammatory synovitis (4–8). Studies examining RA pathogenesis in this early period of disease evolution are limited, but it is hypothesized that macrophages and fibroblasts are key cell types that conspire in this process (9–11). Additionally, cell-to-cell interactions between synovial macrophages and fibroblasts mediate and control inflammatory and fibrotic processes in RA synovium (12, 13). In response to microenvironmental stimuli, macrophages secrete many soluble factors that promote fibroblast differentiation and transformation into an aggressive phenotype (14, 15). In turn, these human fibroblast-like synoviocytes (HFLS) are major effector cells in accelerating extracellular matrix (ECM) destruction and overproduction, facilitating cartilage damage, and promoting expansive synovial tissue or pannus formation (13, 14, 16, 17). Persistent exposure of HFLS cells to the inflammatory milieu likely contributes to their development into an aggressive phenotype, a unique feature of transformed RA HFLSs (16–20). However, the molecular mechanisms and perpetuating microenvironmental factors that elicit a pathogenic phenotype that is consistent with early RA are currently unknown.

Post-translational modifications (PTMs) of macromolecules are known to alter protein conformation, function, and cellular location. Formation of PTMs on proteins can create new binding sites, leading to the formation of novel damage- and/or pathogen-associated molecular patterns (DAMPs/PAMPs) that bind pattern recognition receptors (PRRs) influencing inflammatory responses (21, 22). Prolonged and repeated exposure to PTMs may elicit adaptive immune responses (23–25). Antibodies to modified proteins, in turn, form immune complexes that lead to complement activation and perpetuation of chronic inflammation (26, 27). One such PTM that has been associated with RA is citrullination (28–30). Although citrullination is by itself non-specific, the formation of anti-citrullinated protein antibodies (ACPA) are highly disease-specific (~98%) and can be detected years prior to RA onset (31). Several citrullinated ECM proteins, including fibrinogen, vimentin, and type II collagen have been recognized as ACPA targets (32–36).

Malondialdehyde-acetaldehyde adducts (MAA), by-products of lipid peroxidation, are another PTM that is a target of both innate and adaptive immunity. Anti-MAA antibodies are increased in the serum of RA patients compared to controls and are associated with circulating levels of ACPA (37). As with ACPA, anti-MAA antibodies can be detected years before RA onset, with levels diverging from those of controls on average 2-3 years prior to RA diagnosis (38). MAA-modified proteins are enriched in affected synovial and lung tissues from RA patients. At those sites, MAA strongly co-localizes with citrullinated antigens and mature B cells with correlation coefficients based on imaging techniques that approach 0.9 (39, 40). Highly relevant to this study, MAA-modified proteins elicit robust immune responses in the absence of adjuvant, promoting the formation of autoantibodies and autoreactive T cells (41–44). Exposure to MAA-modified and/or citrullinated fibrinogen alters macrophage phenotype and generates pro-inflammatory and/or pro-fibrotic responses that are unique to the modifications present (45). Together, these studies suggest that MAA adducts act together with citrullinated proteins to initiate and perpetuate early innate immune responses that lead to disease-specific immunity during the pre-clinical phases of RA. However, the cellular responses and potential interactions between macrophages and fibroblasts following exposure to either citrullinated or MAA-modified proteins have not been well delineated.

Given previous observations that MAA-modified and citrullinated proteins are enriched (and colocalize) in RA synovium (37, 39), we hypothesized that macrophages release soluble factor(s) in response to MAA-modified and/or citrullinated fibrinogen that induce fibroblast activation to alter their cellular phenotype and promote ECM deposition/remodeling. To test this hypothesis, we evaluated whether supernatants from activated macrophages affected cellular responses of HFLS derived from RA synovium (HFLS-RA). In addition, soluble factor(s) present in macrophage supernatants were identified and effects on signaling pathways in HFLS-RA cells elucidated.

## 2 Materials and methods

**Figure 1:**
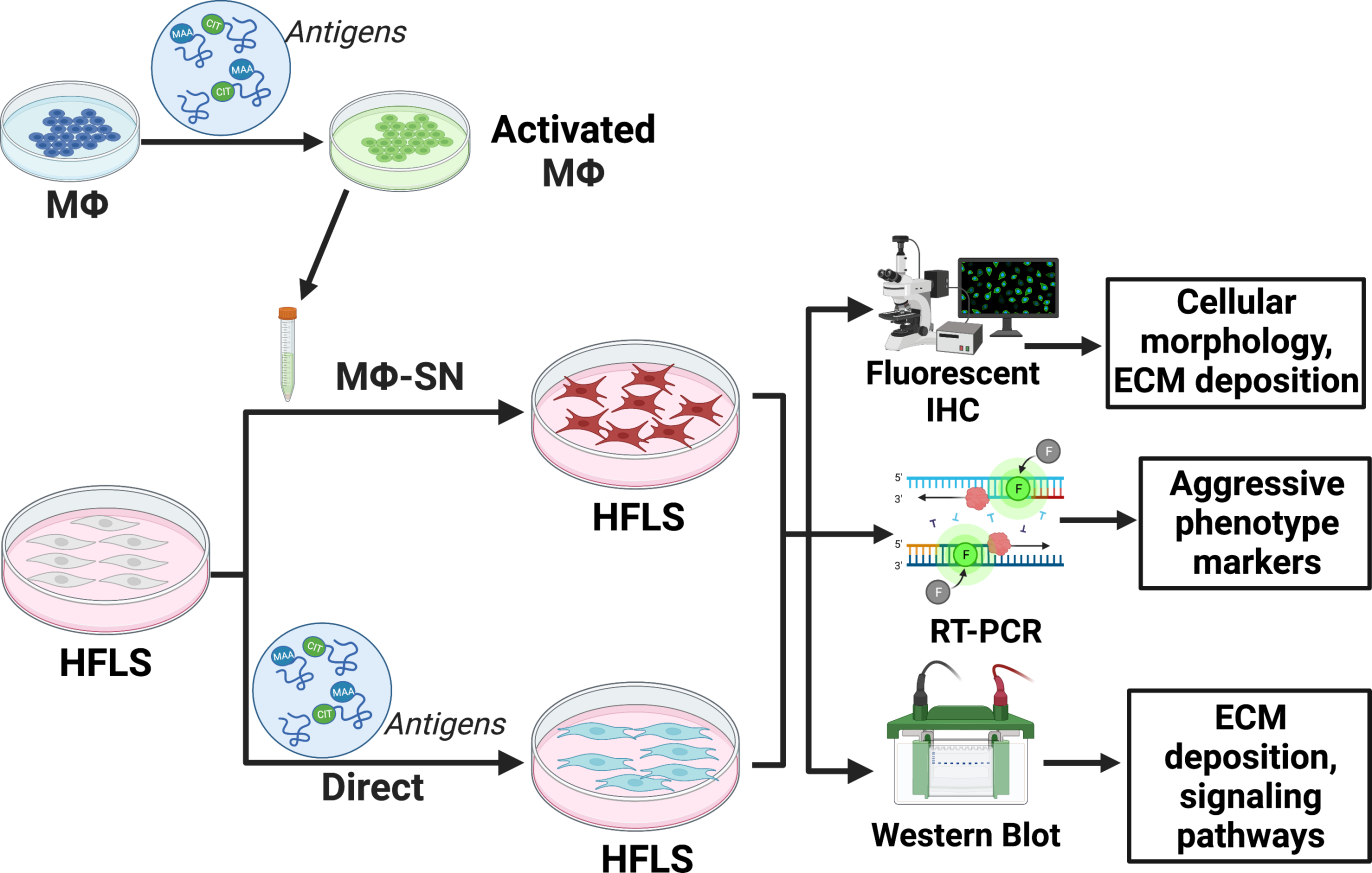
Experimental set-up outlined in the methods section. The figure was created using BioRender. HFLS, human fibroblast like synoviocytes; IHC, immunohistochemistry; Mϕ, macrophages; Mϕ-SN; macrophage supernatants; RT-PCR, real-time polymerase chain reaction. Created with BioRender.com.

**Figure 1** provides a summary of experimental design outlined below.

### 2.1 Preparation of modified fibrinogen

Unmodified or modified forms of fibrinogen (FIB) were used as antigens of interest in this study based on our previous work demonstrating macrophage activation in response to modified FIB antigens (45) and their role in mediating RA-related inflammation (27, 32). MAA modified and/or citrullinated (CIT) FIB antigens were prepared as previously described (45, 46). For MAA modification, native FIB (Cayman Chemical, Ann Arbor, MI, USA) was incubated at a 2:1 ratio of malondialdehyde (2 mM) to acetaldehyde (1 mM) in phosphate buffer (pH 7.2) at 37°C for three days and dialyzed against phosphate buffer for 24-h at 4°C. MAA adduction was confirmed by measuring fluorescence of the dihydropyridine ring structure (excitation 398 nm and emission 460 nm) using a Turner Biosystems (Sunnyvale, CA, USA) LS-5B spectrofluorometer.

For CIT modifications, native or MAA-modified FIB was incubated with rabbit skeletal peptidyl arginine deaminase-2 (PAD2; Sigma Aldrich, Saint Louis, MO, USA) and surplus PAD2 was precipitated out using soy bean trypsin inhibitor (Thermo Fischer, Waltham, MA, USA) (46). CIT modification was verified by ELISA and Western Blotting.

For dual modifications with MAA and CIT, FIB was first MAA- and then CIT-modified based on preliminary data demonstrating optimal cellular responses with modifications generated in this order (46). All modified antigens were sterile filtered through a 0.2µm filter and assessed for endotoxin contamination with levels universally below the detectable limit (46).

### 2.2 Macrophage cell-line culture and stimulation

U-937 cells (human monocytic cell line, ATCC, Manassas, VA, USA) were maintained at 1x10^6^ cells/mL in RPMI medium at 37°C in a 5% CO_2_ incubator. For all experiments, U-937 cells were first differentiated to M0 macrophages (Mϕ) by incubating overnight with 100ng/mL phorbol 12-myristate 13-acetate (PMA) (Sigma Aldrich), followed by a 24-h incubation in RPMI medium. PMA-activated cells were stimulated at 37°C with 25 μg/mL of FIB protein that was unmodified (FIB); MAA-modified (FIB-MAA); citrullinated (FIB-CIT); or MAA-modified and citrullinated (FIB-MAA-CIT). Post 24- and 48-h stimulation, macrophage supernatants (Mϕ-SN) were collected for further evaluation and HFLS cellular stimulations as detailed below.

### 2.3 Monocyte sample collection, isolation, differentiation, and antigen stimulation

Peripheral blood was collected from healthy donors (n=3). Informed consent was obtained from all participating individuals for these studies, which were approved by the University of Nebraska Medical Center Institutional Review Board (IRB#:608-22-EP). Peripheral blood mononuclear cells (PBMCs) were obtained using SepMate™ PBMC isolation tubes as per manufacturer’s protocol (StemCell™ Technologies, Vancouver, Canada) and cultured in RPMI 1640 medium supplemented with 10% fetal bovine serum (FBS), 2mM L-glutamine, 1% penicillin-streptomycin, and 0.05mM 2-mercaptoethanol. PBMCs were then differentiated into M0 macrophages (MP) using 100ng/mL of macrophage colony stimulating factor (M-CSF; Thermo Fischer) for 7 days (47). These differentiated macrophages were stimulated with unmodified and modified FIB antigens as outlined above. At 24- and 48-h, the supernatants (MP-SN) were collected for subsequent multiplex immunoassay analysis and HFLS cellular stimulations as outlined below.

### 2.4 HFLS cell culture and stimulations

Primary HFLS cells from healthy controls (HFLS control), osteoarthritis synovium (HFLS-OA), and RA synovium (HFLS-RA) were purchased from Cell Applications, Inc (San Diego, CA, USA). HFLS cells were grown to confluency in Synoviocyte Growth Medium (Cell Applications). Two independent sets of stimulations were performed on HFLS cells as outlined below.

#### 2.4.1 Stimulation of HFLS cells with macrophage supernatants

To investigate whether soluble factors are released from macrophages that affect fibroblast function, HFLS cells were stimulated with Mϕ-SN (U-937 cell line) or MP-SN (isolated human monocytes) collected post-treatment of Mϕ with unmodified and modified FIB antigens. Supernatants from isolated human PBMCs (MP-SN) that were stimulated with the same FIB antigens were utilized to verify whether observations using Mϕ-SN from the U-937 cell lines were similar to those in human macrophages. HFLS cells were stimulated with the standard volumes of Mϕ-SN or MP-SN (25% of total well volume).

#### 2.4.2 Direct stimulation of HFLS cells

Since the Mϕ-SN or MP-SN contain residual FIB antigens, HFLS cells were directly stimulated with unmodified and modified FIB as described for macrophage simulations above in order to account for the direct effects that any residual antigens present in the supernatants may have on HFLS cells.

### 2.5 RNA isolation and real-time PCR analysis

RT-PCR was used to evaluate the effects of Mϕ-SN (or MP-SN) vs. direct antigen stimulations on HFLS cells. This approach was chosen as experiments using Mϕ-SN or MP-SN are confounded by the presence of soluble proteins in supernatants, prohibiting the attribution of cell source (Mϕ vs. HFLS) for these analytes. Therefore, we focused these studies on the measurement of gene expression rather than protein to minimize such confounding and to facilitate interpretation. RNA from HFLS cells was extracted using RNeasy® Mini Kit (Qiagen, Hilden, Germany), quantified using Nanodrop 2000c (Thermo Fischer). HFLS gene expression was assessed for 763 genes up to 8-h post-stimulation (as detailed above) using the Nanostring® Human Fibrosis Panel (Nanostring Technologies, Seattle, WA, USA). The 8 genes with the highest mRNA fold increase vs. FIB were then evaluated using real-time (RT)-PCR at 8- and 24-h post-stimulation of HFLS cells. For RT-PCR, extracted RNA was reverse transcribed to cDNA using a High-capacity RNA-to-cDNA™ kit (Applied Biosystems, Waltham, MA, USA). The following primers for the 8 genes (*italicized* below) were used in RT-PCR analysis; *interleukin (IL)-1β, IL-6, type II collagen (COL2A1), metalloproteinase (MMP)-9, MMP-10, MMP-12, transforming growth factor (TGF)-β*, and *vimentin (VIM)*. The genes were categorized based on their role in fibroblast activation as pro-inflammatory (*IL-1β, IL-6)*; pro-fibrotic (*TGF-β, VIM)*; pro-invasive (*MMP-9, MMP-10, MMP-12)*; and pro-chondrogenesis *(COL2A1)* (16, 48–54). The amplification reaction assays contained cDNA, TaqMan™ Gene Expression Master Mix (Applied Biosystems), and Fluorescein Amidite (FAM) tagged primers (Applied Biosystems). The 7500 Fast RT-PCR System (Applied Biosystems) software was used for amplification and analysis. Data were normalized to native FIB and presented as relative mRNA quantity (Rq). Examination of these soluble proteins (IL-1β, IL-6, MMP-9, MMP-10, MMP-12, TGF-β) was not performed due to interference/confounding of the soluble proteins present in Mϕ-SN or MP-SN as outlined above. In addition, extracellular matrix protein deposition of vimentin (gene: *VIM*) and type II collagen (gene: *COL2A1*) were examined further as outlined below, as these are only deposited from HFLS cells. Stimulation with media alone served as a negative control and demonstrated negligible upregulation of the genes examined (data not shown).

### 2.6 Detection of extracellular matrix proteins using fluorescent immunohistochemistry

To quantify ECM protein deposition and cell-culture morphological changes following stimulation with unmodified and modified FIB, formalin-fixed HFLS cells were examined using fluorescent immunohistochemical (IHC) staining for the presence of type II collagen and vimentin. Although, type II collagen is recognized to be primarily found in cartilage, others have reported expression of this osteochondrogenic marker in aggressive phenotype of synovial fibroblasts and was thus included in these experiments (51, 52, 55). Additionally, vimentin is well recognized as an intracellular scaffold protein, it is deposited into the ECM under pathologic conditions such as those posed by RA (49, 56, 57) and was thus included in these experiments.

Briefly, HFLS cells were cultured in 24-well flat-bottom glass plates (Cellvis, Mountain View, CA, USA). The cells were then incubated for 48-h with unmodified/modified FIB antigens or Mϕ-SN as described above. Stimulation with media alone served as a negative control and demonstrated negligible effects on expression of ECM proteins (data not shown). Cells were then washed with PBS, blocked with 2% goat serum, and incubated overnight with a rabbit polyclonal Alexa Flour 594-conjugated antibody to collagen II (Bioss, Woburn, MA, USA) or a polyclonal rabbit Cy5-conjugated antibody to vimentin (Bioss). The cells were again washed, incubated with DAPI for nuclear staining for 2 minutes, mounted in fluoromount-G® (SouthernBiotech, Birmingham, AL, USA), and subjected to imaging using a Zeiss 710 Meta confocal laser-scanning microscope. Native FIB treated cells were used to determine microscope settings and all treatment group images were acquired using these identical parameters. Images were analyzed using Zen 2012 software (Zeiss, Jena, Germany) and quantified as mean pixel density (MPD) using ImageJ (National Institutes of Health, Bethesda, MD, USA).

### 2.7 Wound scratch assay

HFLS-RA cells were cultured in a 6-well plate at 37°C and 5% CO_2_ until reaching 90% confluency. The confluent cell monolayer was scratched with 200 μL sterile pipette tip followed by washing of cells with PBS to remove nonadherent cells. Thereafter, cells were treated with either unmodified/modified FIB antigens or Mϕ-SN as described above. The images were captured at t = 0h to record the initial area of the scratch and at t = 24h to evaluate effects of different treatments. Cell migration was monitored by collecting digitized images and 24 hours was selected as our experimental time point as this timeframe provided sufficient cell migration for detecting biological differences without entirely filling up the scratched area. Digitalized images were captured using the light microscopy setting on a Zeiss 710 Meta microscope. Images were analyzed using Image J software to measure the scratched or decellularized area (A). Cell migration was quantified as wound confluence using the following calculation (58): Wound confluence = [A(t_0_) – A(t_24_)]/[A(t_0_)].

### 2.8 Western blot analysis

#### 2.8.1 Detection of extracellular matrix proteins in HFLS cells

As IHC studies demonstrated increased HFLS-mediated vimentin and type II collagen protein deposition, additional studies were undertaken to quantify protein expression. In brief, HFLS cells were stimulated with unmodified/modified FIB antigens or Mϕ-SN as above for 48-h. Cellular lysates were prepared using radioimmunoprecipitation assay (RIPA) buffer (Thermo Fisher) in the presence of protease inhibitor cocktail (Roche, Basel, Switzerland) (45). The bicinchoninic acid (BCA) assay (Thermo Fisher) was performed to measure protein concentrations of lysates. Cellular lysates were subjected to SDS-PAGE electrophoresis and transferred to a polyvinylidene difluoride (PVDF) membrane (EMD Millipore Corp, Burlington, MA, USA) for Western Blot analysis. PVDF membranes were blocked in 2% casein (Sigma Aldrich) and incubated with the following primary antibodies overnight; a monoclonal (V9) mouse anti-vimentin IgG antibody (Abcam, Boston, MA, USA), a monoclonal (5B2.5) anti-collagen type II antibody (Novus Biologicals, Centennial, CO, USA), a polyclonal rabbit anti-collagen type II antibody (EMD Millipore Corp), and a polyclonal rabbit anti-β-actin IgG antibody (endogenous control) (Novus Biologicals). The following day, blots were incubated with HRP-conjugated secondary antibodies (Jackson ImmunoResearch, West Grove, PA, USA): goat anti-mouse IgG or goat anti-rabbit IgG. Chemiluminescent reagent (Thermo Fisher) was utilized for the blot development and visualization using the KwikQuant imager (Kindle Biosciences LLC, Greenwich, CT, USA). Respective band intensities were measured using KwikQuant Image Analyzer (version 1.8.6) and reported as mean band intensities normalized to β-actin.

#### 2.8.2 Activation of cell signaling pathways in HFLS cells

Due to unique cellular responses observed in HFLS-RA cells (compared to HFLS-OA or HFLS control cells), cell signaling pathways were examined only in HFLS-RA cells. Several signal transduction pathways involved in the transformation of HFLS-RA cells to an aggressive phenotype were examined (16, 50, 59). Briefly, HFLS cells were incubated with antigens for 15-min, 30-min, 1-h and 4-h, cellular lysates were prepared as above, and the following primary antibodies (all polyclonal rabbit IgGs) were utilized to probe the samples; anti-β-actin antibody (endogenous control) (Novus Biologicals), anti-SAPK/JNK (#9252), anti-phospho-SAPK/JNK (Thr183/Tyr185) (#9251), anti-Erk1/2 (p44/42 MAPK) (#9102), anti-phospho-Erk1/2 (p44/42 MAPK; Thr202/Tyr204) (#9101), anti-Akt (#9272), anti-phospho-Akt (Ser473) (#9271), anti-p38 MAPK (#9212), anti-phospho-p38 MAPK (Thr180/Tyr182) (#4511), anti-Paxillin (#2542), anti-phospho-Paxillin (Tyr118) (#2541), anti-FAK (#3285), anti-phospho-FAK (Tyr397) (#3283) (Cell Signaling Technology, Danvers, MA, USA). HRP-conjugated anti-rabbit IgG (Jackson ImmunoResearch) was used as a secondary antibody. Respective band intensities were measured and reported as outlined above. The maximum and minimum activation of signaling pathways for HFLS lysates was determined based on positive (treatment with LPS) and negative (treatment with media) controls.

### 2.9 Measurement of platelet derived growth factor-BB

Given that the phosphorylated signaling pathways were similar to that reported by others following stimulation with platelet derived growth factor (PDGF)-BB (60–63), additional studies were undertaken to quantify this analyte in Mϕ-SN and MP-SN. Mϕ-SN (U-937 derived) and MP-SN (PBMC-derived) were assayed for the secretion of PDGF-BB isoform using a commercial immunoassay platform (Meso Scale Discovery, Rockville, MD, USA; lower limit of detection of 0.29 pg/ml). PDGF-BB isoform was examined in these studies due to its role in RA pathogenesis and activation of signaling mechanisms in fibroblasts as observed in other studies (64, 65). The assays were performed according to the manufacturer’s protocol and analyzed on the Meso QuickPlex SQ 120 imager (Meso Scale Discovery).

### 2.10 Stimulation of HFLS cells with PDGF-BB

Detection of PDGF-BB in Mϕ-SN led to a separate set of experiments to assess its effects on HFLS-RA cells. The cells were stimulated with either 10ng/mL or 5pg/mL of PDGF-BB (Thermo Fisher). Post-stimulation, HFLS-RA cells were examined for ECM protein expression using fluorescent IHC and for activation of signaling pathways using Western Blot as described above.

### 2.11 Statistical analyses

All statistical analyses were completed using GraphPad Prism 9.0.0 Software (San Diego, CA, USA). Normality was confirmed using Shapiro-Wilk test (for n>3). Experimental data are presented as the mean ± standard error of the mean (SEM) based on ≥ 3 replicate experiments. Statistical differences among groups were completed using one-way analysis of variance (ANOVA) with Tukey’s *post-hoc* test (for n>3) or the Kruskal-Wallis test with Dunn’s multiple comparisons test (for n=3). Mann Whitney non-parametric test (for n=3) was used for comparisons between two treatment groups (MΦ-SN vs. direct stimulation). Differences were considered statistically significant when P values were less than 0.05.

## 3 Results

### 3.1 Upregulation in the expression of genes associated with an aggressive phenotype in HFLS cells stimulated with Mϕ-SN vs. direct antigen stimulation

Mϕ-SN collected post 48-h stimulation demonstrated greater mRNA fold change in HFLS-RA markers compared to 24-h collection of Mϕ-SN (data not shown). Hence Mϕ-SN collected at 48-h were used for all HFLS cell stimulations.

**Figure 2:**
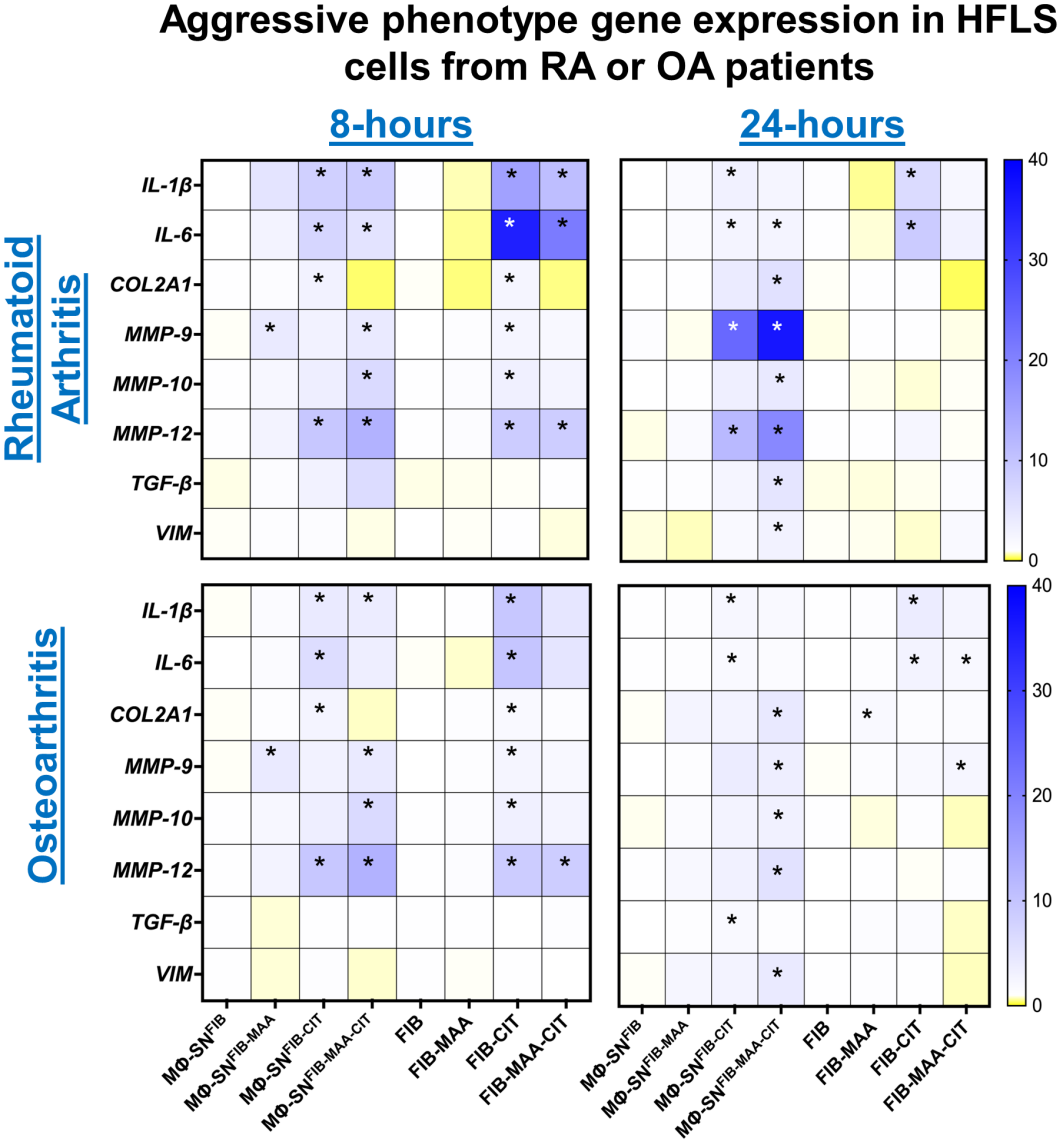
Heatmap of RT-PCR demonstrating expression of genes associated with an aggressive cell phenotype in stimulated HFLS-RA and HFLS-OA cells. HFLS cells were treated with macrophage supernatants (Mϕ-SN) or direct antigen stimulation for 8- and 24- hours. Mϕ-SN were collected post-treatment of PMA-activated U-937 cells with the modified fibrinogen (FIB) antigens. Direct stimulation corresponds to treatment of HFLS cells with unmodified and modified FIB. Pro-inflammatory genes: *IL-1β, IL-6*. Pro-fibrotic genes: *TGF-β, VIM.* Pro-invasiveness genes*: MMP-9, MMP-10, MMP-12. Pro-chondrogenesis gene: COL2A1*. Data are shown as a mean of relative quantity (Rq) with standard error of the mean (SEM) of markers. Blue boxes represent increased mRNA levels, while yellow boxes represent decreased mRNA levels. Gradient transition shows lowest to highest expression by color compared to FIB or Mϕ-SN^FIB^ (Rq=1.0) represented as white color. Kruskal-Wallis non-parametric test with *post-hoc* Dunn’s multiple comparison test are shown when treatment groups were compared to Mϕ-SN^FIB^ or FIB, *p<0.05, n=3. COL2A1, pro-alpha1 chain of type II collagen, FIB, native fibrinogen; FIB-MAA, MAA-modified fibrinogen; FIB-CIT, citrullinated fibrinogen; FIB-MAA-CIT, MAA and citrulline modified fibrinogen; HFLS, human fibroblast-like synoviocytes; IL-interleukin; MMP, metalloproteinase; Mϕ-SN; macrophage supernatants; TGF-β, transforming growth factor beta; VIM, vimentin.

To examine the effects of Mϕ-SN and direct antigens on HFLS cells derived from patients with rheumatoid arthritis (HFLS-RA), genetic markers of an aggressive phenotype were evaluated at 8-h time point. After 8-h, stimulation of HFLS-RA with Mϕ-SN^FIB-CIT^, and Mϕ-SN^FIB-MAA-CIT^ led to significant mRNA upregulation of the pro-inflammatory genes IL-1*β* and *IL-6* (2- to 7-fold higher vs. Mϕ-SN^FIB^; p<0.05) (**Figure 2**). Additionally, expression of pro-invasiveness genes *MMP-9, MMP-10* and *MMP-12* were also significantly increased between 2- to 10-fold (p<0.05) following stimulation with Mϕ-SN^FIB-MAA-CIT^, which universally yielded the highest mRNA fold increase (**Supplemental Figure 1**). In contrast, direct stimulation of HFLS-RA with FIB-CIT promoted the highest mRNA fold increase for pro-inflammatory cytokines (*IL-1β*: 12-fold, p<0.001; *IL-6*: 26-fold, p<0.001 vs. FIB). Additionally, direct stimulation of HFLS-RA with FIB-CIT and FIB-MAA-CIT promoted increases in mRNA levels for pro-invasiveness and pro-chondrogenesis genes that were lower in magnitude than those observed following stimulation with Mϕ-SN. This included increases in mRNA for *MMP-9* (FIB-CIT: 2-fold, p<0.05), *MMP-10* (FIB-CIT: 3-fold, p<0.05), and *MMP-12* (FIB-CIT: 7-fold, p<0.05; FIB-MAA-CIT: 7-fold, p<0.05) compared to FIB. Exposure of HFLS-RA to FIB-MAA did not significantly change mRNA levels of the genes. Similar expression patterns were observed in HFLS cells derived from patients with osteoarthritis (HFLS-OA) with smaller mRNA fold changes for the above markers (**Figure 2**; **Supplemental Figure 2**).

To evaluate durability of responses observed after 8-h, additional measurements were completed following 24-h stimulation. After 24-h, *COL2A1*, *MMP-9, MMP-10, MMP-12, TGF-β, and VIM* genes were significantly upregulated, between 4- to 35-fold (p < 0.05) following stimulation of HFLS-RA with Mϕ-SN^FIB-MAA-CIT^. Further, stimulation of HFLS-RA with Mϕ-SN^FIB-MAA-CIT^ resulted in the highest fold increases of aggressive phenotype genes based on expression of *MMP-9, MMP-12, TGF-β*, and *VIM* (**Figure 2**; **Supplemental Figure 1**). The relative quantity of mRNA levels for the pro-inflammatory genes related to Mϕ-SN^FIB-CIT^ and Mϕ-SN^FIB-MAA-CIT^ stimulation remained elevated, but at a lower magnitude compared to values following 8-h, suggesting a decrease in the inflammatory response following an initial increase. Of note, direct stimulation of HFLS-RA at 24-h yielded smaller fold-changes on mRNA expression for all of the markers in comparison to 8-h. Further, only pro-inflammatory genes remained significantly upregulated in HFLS-RA cells after exposure to FIB-CIT as compared to the 8-h time point. Similar observations were made at 24-h for HFLS-OA cells (**Figure 2**; **Supplemental Figure 2**). Together, these data suggest that HFLS-RA cells exposed to Mϕ-SN^FIB-MAA-CIT^ sustained gene upregulation of aggressive phenotype as compared to direct antigen stimulation. Additionally, these responses were lower in magnitude in HFLS-OA cells.

**Figure 3:**
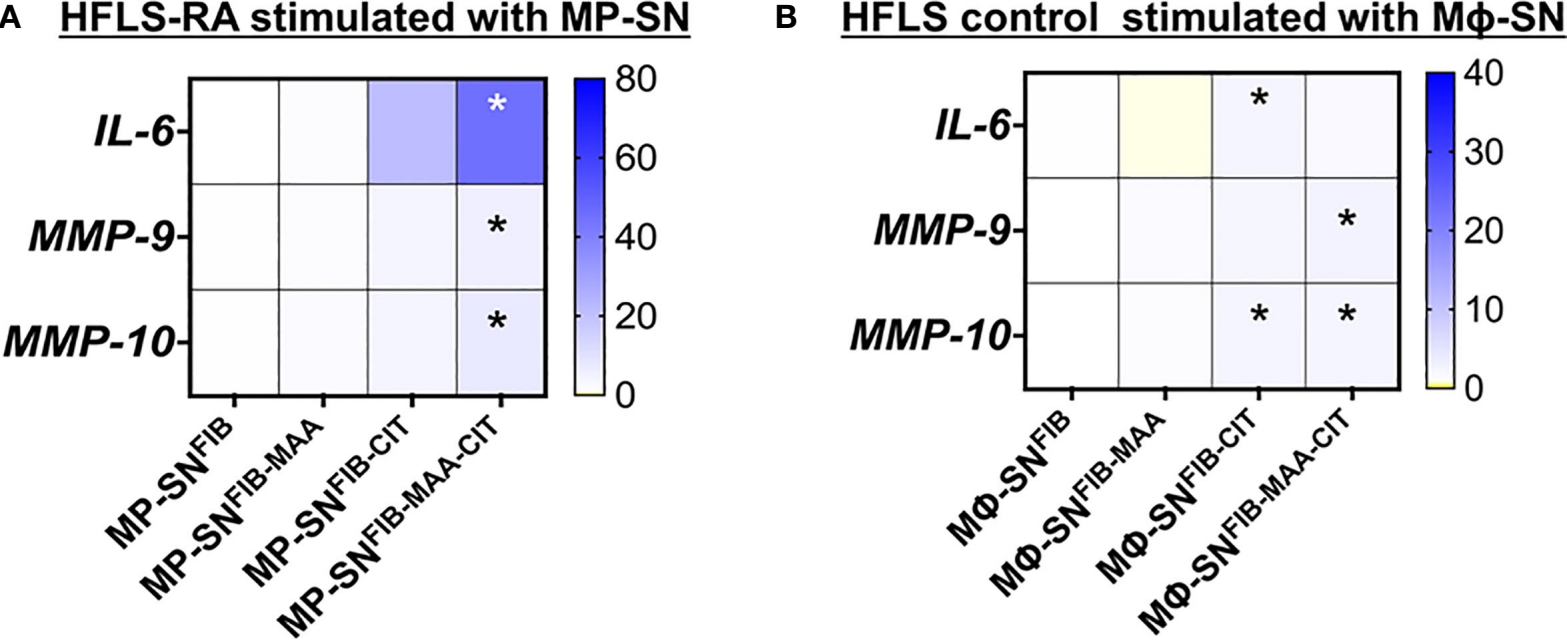
Heatmaps of RT-PCR for markers in stimulated HFLS cells. **(A)** HFLS-RA cells were treated with MP-SN for 8 hours. MP-SN corresponds to treatment of HFLS-RA cells with PBMC supernatants (MP-SN) from PBMC stimulation with the modified fibrinogen antigens. **(B)** HFLS control cells were treated with Mϕ-SN for 8 hours. HFLS control cells were treated with Mϕ-SN collected post-treatment of U-937 cells with the modified fibrinogen antigens. Blue boxes represent increased mRNA levels, while yellow boxes represent decreased mRNA levels. Gradient transition shows lowest to highest expression by color with baseline (Rq=1.0) represented as white color. Kruskal-Wallis non-parametric test with *post-hoc* Dunn’s multiple comparison test are shown when treatment groups were compared to Mϕ-SN^FIB^ or FIB, *p<0.05, n=3. FIB, native fibrinogen; FIB-MAA, MAA-modified fibrinogen; FIB-CIT, citrullinated fibrinogen; FIB-MAA-CIT, MAA and citrulline modified fibrinogen; IL-interleukin; MMP, metalloproteinase.

In separate experiments to determine whether freshly isolated human PBMCs reacted similarly to the U-937 cell line utilized above, HFLS-RA cells were stimulated with MP-SN activated with unmodified or modified FIB antigens and showed analogous increases in mRNA levels for *IL-6*, *MMP-9*, and *MMP-10* as observed following HFLS-RA cell stimulation with the Mϕ-SN (U-937 derived Mϕ-SN) (**Figure 3A**; **Supplemental Figure 3A**). Additionally, HFLS control cells from healthy synovium were used as a baseline control cell line. HFLS control cells amplified mRNA levels for *IL-6*, *MMP-9*, and *MMP-10* in response to treatment with Mϕ-SN^FIB-CIT^ and Mϕ-SN^FIB-MAA-CIT^, but at smaller fold changes compared to HFLS-RA cells (**Figure 3B**; **Supplemental Figure 3B**). *IL-6* (pro-inflammatory gene) and *MMP-9* (pro-invasiveness gene) were chosen for these studies due to the highest Rq values in HFLS-RA cells, while MMP-10 (pro-invasiveness gene) was chosen based on the moderate (range from 3- to 5- fold) Rq amplification pattern.

### 3.2 Exposure of HFLS-RA cells to Mϕ-SN promotes aggressive phenotype by increasing extracellular matrix deposition, unique morphological changes, and invasiveness

**Figure 4:**
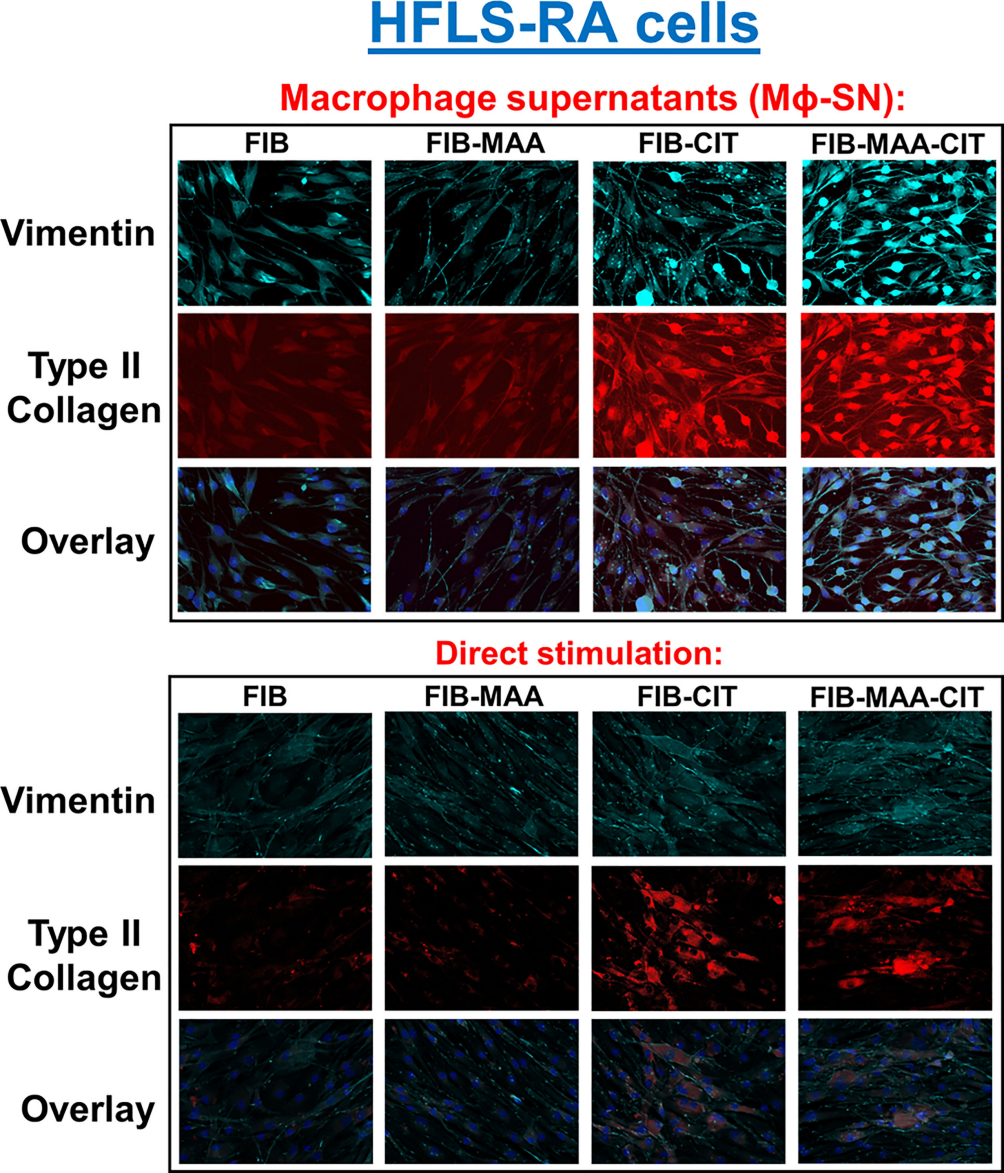
Fluorescent immunohistochemistry (IHC) of extracellular matrix (ECM) proteins in stimulated HFLS-RA cells. HFLS-RA cells were treated with macrophage supernatants (Mϕ-SN) or direct antigen stimulation. Mϕ-SN were collected post-treatment of PMA-activated U-937 cells with the modified fibrinogen (FIB) antigens. Direct stimulation corresponds to treatment of HFLS-RA cells with the modified FIB antigens. Fluorescent IHC images of HFLS-RA cells stained for vimentin and type II collagen. N=6. FIB, native fibrinogen; FIB-MAA, MAA-modified fibrinogen; FIB-CIT, citrullinated fibrinogen; FIB-MAA-CIT, MAA and citrulline modified fibrinogen; Mϕ-SN; macrophage supernatants.

**Figure 5:**
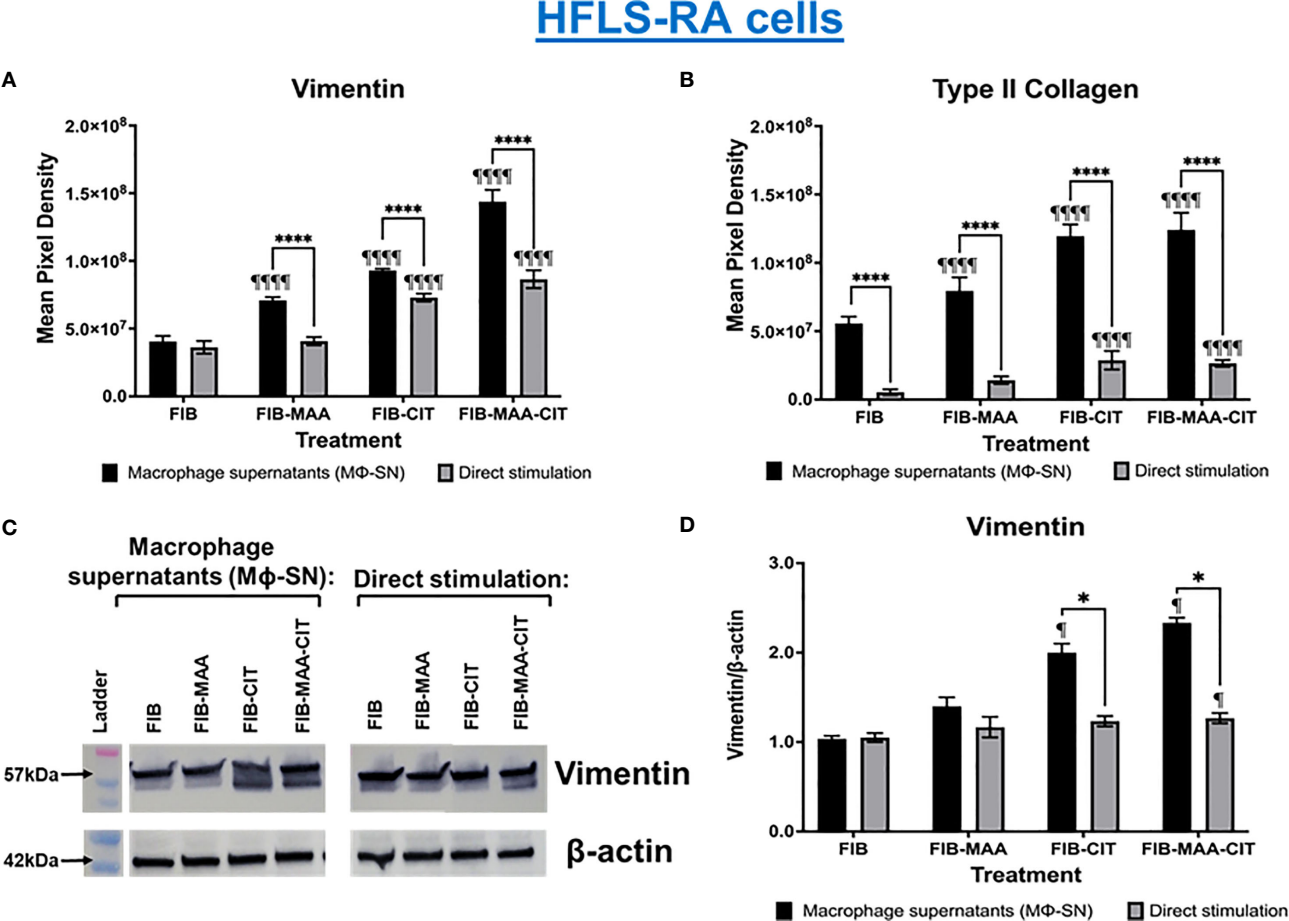
Quantification of fluorescent IHC and Western Blot of ECM proteins in stimulated HFLS-RA cells. HFLS-RA cells were treated with macrophage supernatants (Mϕ-SN) or direct antigen stimulation. Mϕ-SN were collected post-treatment of PMA-activated U-937 cells with the modified fibrinogen (FIB) antigens. Direct stimulation corresponds to treatment of HFLS-RA cells with the modified FIB antigens. Quantification of fluorescent IHC images measuring mean pixel density for vimentin **(A)** and type II collagen **(B)**. **(C)** HFLS-RA lysates probed with anti-vimentin and anti-β-actin antibodies. **(D)** Densitometry of normalized values to β-actin of vimentin. The data is represented as a mean of meal pixel density (MPD) with SEM for Fluorescent IHC and as a mean of densitometry normalized values with SEM for Western Blot. For IHC, comparisons made to Mϕ-SN^FIB^ or FIB are illustrated above each bar: ^¶¶¶¶^p<0.0001, ^¶^p<0.05; n=6. One-way ANOVA with Tukey’s *post-hoc* test comparisons are illustrated between treatment groups, and only significant differences are illustrated: ****p<0.0001, *p<0.05. For Western Blot, Kruskal-Wallis non-parametric test with *post-hoc* Dunn’s multiple comparison test to Mϕ-SN^FIB^ or FIB are illustrated above each bar: ^¶^p<0.05; n=3. Mann Whitney non-parametric test are shown when treatment groups were compared between Mϕ-SN and direct stimulation, *p<0.05, n=3. FIB, native fibrinogen; FIB-MAA, MAA-modified fibrinogen; FIB-CIT, citrullinated fibrinogen; FIB-MAA-CIT, MAA and citrulline modified fibrinogen; Mϕ-SN; macrophage supernatants.

In subsequent studies, we investigated the effect of the same HFLS stimulations on changes in cell morphology in addition to quantifying the cellular production of ECM proteins using both fluorescent immunohistochemistry (IHC) and Western Blot. Compared to HFLS-RA cells directly stimulated with FIB-CIT and FIB-MAA-CIT, HFLS-RA cells stimulated with Mϕ-SN^FIB-CIT^, Mϕ-SN^FIB-MAA-CIT^ demonstrated an elongated and round, “spindle-shaped” morphology (**Figure 4**) in addition to significant increases in the deposition of vimentin (p<0.0001 for Mϕ-SN^FIB-CIT^, p<0.0001 for Mϕ-SN^FIB-MAA-CIT^ vs. direct stimulation with the same antigens) and type II collagen (p<0.0001 for Mϕ-SN^FIB-CIT^, p<0.0001 for Mϕ-SN^FIB-MAA-CIT^) (**Figures 5A, B**). Changes observed in cellular morphology and in the deposition of vimentin were highest following stimulation of HLFS-RA cells with Mϕ-SN^FIB-MAA-CIT^ (p<0.0001 vs. other antigens for vimentin). Stimulation with Mϕ-SN^FIB-MAA^ significantly increased HFLS-RA cellular deposition of vimentin (p<0.0001) and type II collagen (p<0.0001) compared to direct stimulation with the same antigen although yielding less striking changes in cellular morphology. By Western Blot, HFLS-RA cells demonstrated a similar pattern of expression to fluorescent IHC with higher expression of vimentin in treatments with Mϕ-SN compared to direct antigens (p<0.05 for Mϕ-SN^FIB-CIT^, Mϕ-SN^FIB-MAA-CIT^; **Figures 5C, D**). Together these data suggest a unique HFLS-RA cellular morphology and increased protein deposition of vimentin and type II collagen in response to Mϕ-SN. Also, this aligns with RT-PCR data above demonstrating an increase in mRNA levels for vimentin (*VIM*) and type II collagen (*COL2A1*) genes.

**Figure 6:**
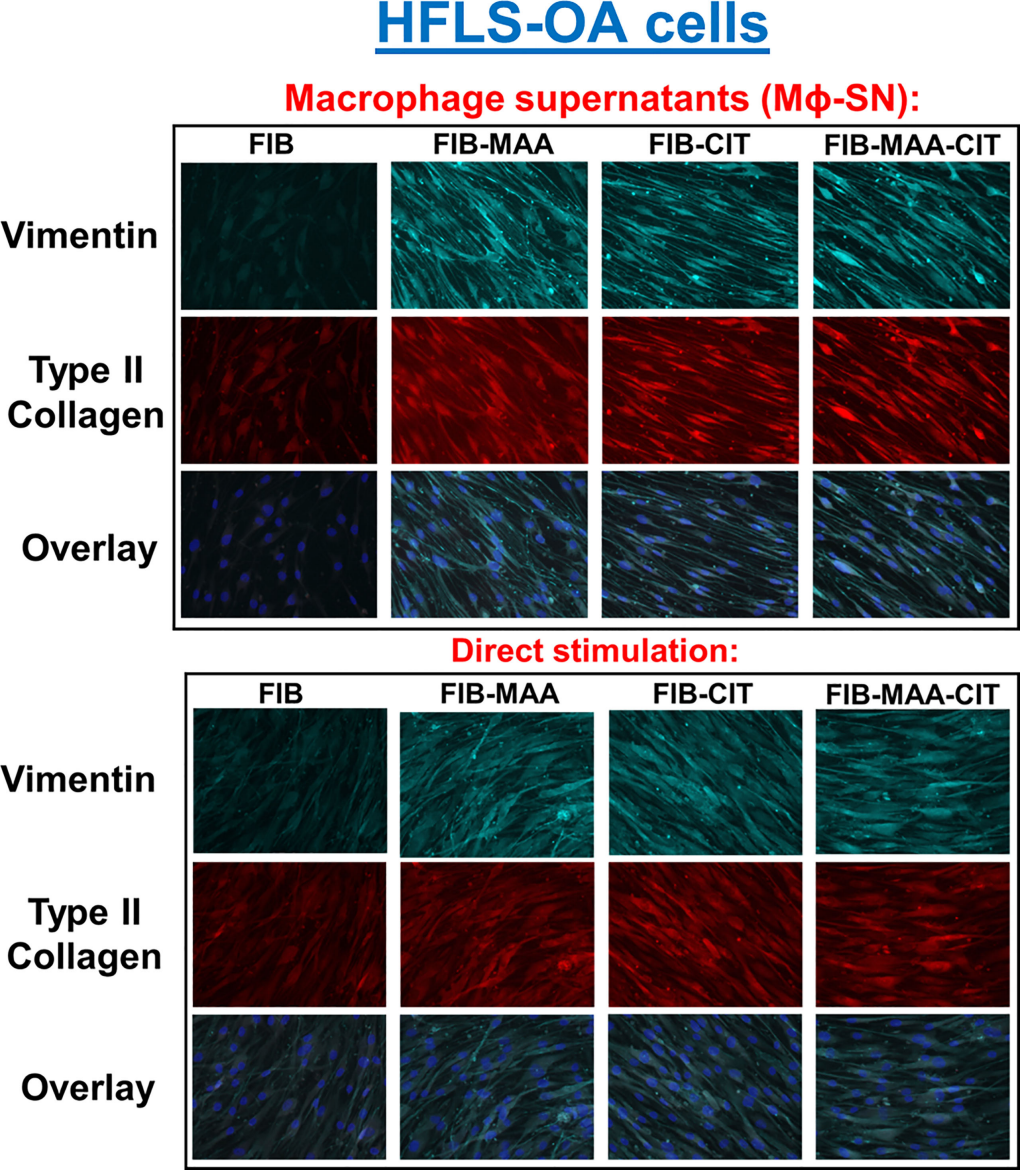
Fluorescent immunohistochemistry (IHC) of extracellular matrix (ECM) proteins in stimulated HFLS-OA cells. HFLS-OA cells were treated with macrophage supernatants (Mϕ-SN) or direct antigen stimulation. Mϕ-SN were collected post-treatment of PMA-activated U-937 cells with the modified fibrinogen (FIB) antigens. Direct stimulation corresponds to treatment of HFLS-OA cells with the modified FIB antigens. Fluorescent IHC images of HFLS-OA cells stained for vimentin and type II collagen. N=6. FIB, native fibrinogen; FIB-MAA, MAA-modified fibrinogen; FIB-CIT, citrullinated fibrinogen; FIB-MAA-CIT, MAA and citrulline modified fibrinogen; Mϕ-SN; macrophage supernatants.

**Figure 7:**
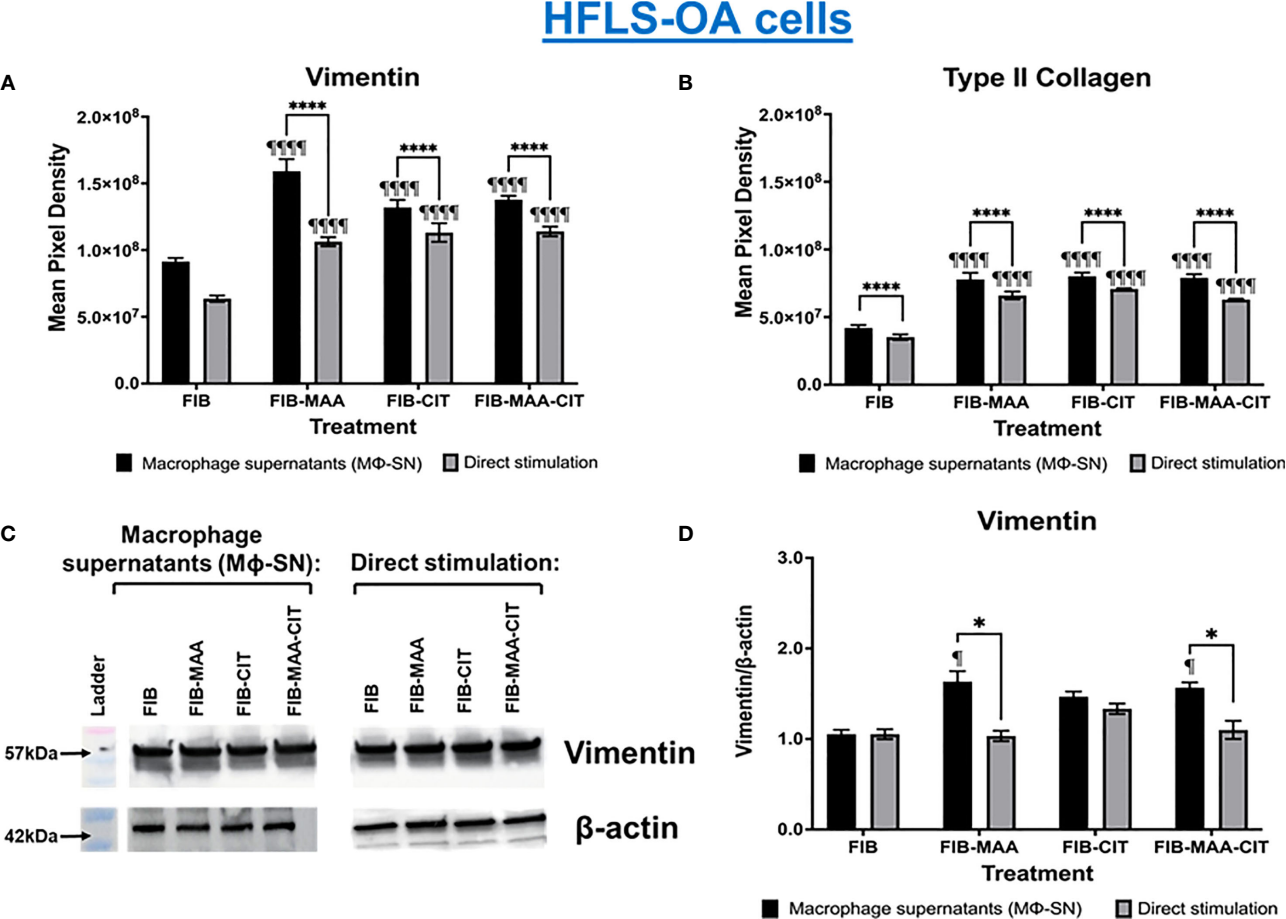
Quantification of fluorescent IHC and Western Blot of ECM proteins in stimulated HFLS-OA cells. HFLS-OA cells were treated with macrophage supernatants (Mϕ-SN) or direct antigen stimulation. Mϕ-SN were collected post-treatment of PMA-activated U-937 cells with the modified fibrinogen (FIB) antigens. Direct stimulation corresponds to treatment of HFLS-OA cells with the modified FIB antigens. Quantification of fluorescent IHC images measuring mean pixel density for vimentin **(A)** and type II collagen **(B)**. **(C)** HFLS-OA lysates probed with anti-vimentin and anti-β-actin antibodies. **(D)** Densitometry of normalized values to β-actin of vimentin. The data is represented as a mean of meal pixel density (MPD) with SEM for Fluorescent IHC and as a mean of densitometry normalized values with SEM for Western Blot. For IHC, comparisons made to Mϕ-SN^FIB^ or FIB are illustrated above each bar: ^¶¶¶¶^p<0.0001, ^¶^p<0.05; n=6. One-way ANOVA with Tukey’s *post-hoc* test comparisons are illustrated between treatment groups, and only significant differences are illustrated: ****p<0.0001, *p<0.05. For Western Blot, Kruskal-Wallis non-parametric test with *post-hoc* Dunn’s multiple comparison test to Mϕ-SN^FIB^ or FIB are illustrated above each bar: ^¶^p<0.05; n=3. Mann Whitney non-parametric test are shown when treatment groups were compared between Mϕ-SN and direct stimulation, *p<0.05, n=3. FIB, native fibrinogen; FIB-MAA, MAA-modified fibrinogen; FIB-CIT, citrullinated fibrinogen; FIB-MAA-CIT, MAA and citrulline modified fibrinogen; Mϕ-SN, macrophage supernatants.

To examine specificity of HFLS responses, the above experiments were then repeated using HFLS-OA cells. Unlike HFLS-RA, stimulation in HFLS-OA with Mϕ-SN did not result in changes in cellular morphology (**Figure 6**). Stimulation of HFLS-OA cells with Mϕ-SN (following stimulation with modified FIB) also increased vimentin and type II collagen expression in comparison to direct antigen stimulation (**Figure 7**). Type II collagen was probed in both HFLS-RA and HFLS-OA lysates with two different antibodies, polyclonal and monoclonal antibodies, and both antibodies to type II collagen were undetectable by Western Blot (data not shown).

**Figure 8:**
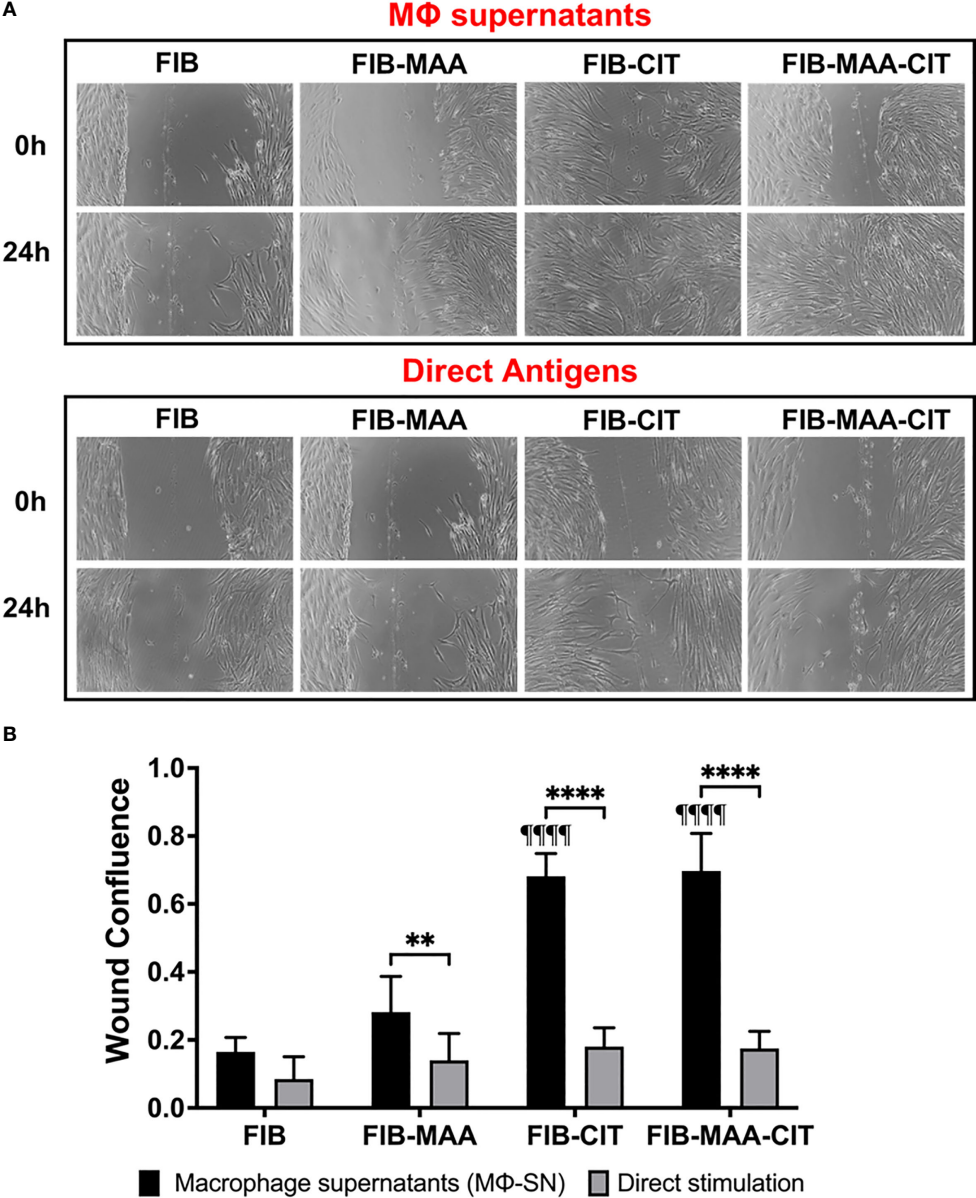
Wound Scratch Assay in stimulated HFLS-RA cells. HFLS-RA cells were treated with macrophage supernatants (Mϕ-SN) or direct antigen stimulation. Mϕ-SN were collected post-treatment of PMA-activated U-937 cells with the modified fibrinogen (FIB) antigens. Direct stimulation corresponds to treatment of HFLS-RA cells with the modified FIB antigens. **(A)** Representative images of wound scratch assay evaluating scratch area at 0h and 24h. **(B)** Quantification of wound closure by calculating wound confluence based on the differences in the scratch area. Comparisons made to Mϕ-SN^FIB^ or FIB are illustrated above each bar: ^¶¶¶¶^p<0.0001; n=6. One-way ANOVA with Tukey’s *post-hoc* test comparisons are illustrated between treatment groups, and only significant differences are illustrated: ****p<0.0001, **p<0.01. FIB, native fibrinogen; FIB-MAA, MAA-modified fibrinogen; FIB-CIT, citrullinated fibrinogen; FIB-MAA-CIT, MAA and citrulline modified fibrinogen; Mϕ-SN; macrophage supernatants.

To confirm the effect of Mϕ-SN on inducing aggressive phenotype in HFLS-RA cells, cellular migration was evaluated using the wound scratch assay. Stimulation of HFLS-RA with Mϕ-SN^FIB-CIT^ and Mϕ-SN^FIB-MAA-CIT^ promoted the highest wound confluence 0.68 ±0.02 and 0.69 ±0.05, respectively. Treatment of HFLS-RA with Mϕ-SN from the modified FIB antigens promoted higher wound confluence compared to direct antigens (p<0.05 for all; **Figure 8**).

### 3.3 Activation of JNK, Erk1/2, and Akt signaling pathways

**Figure 9:**
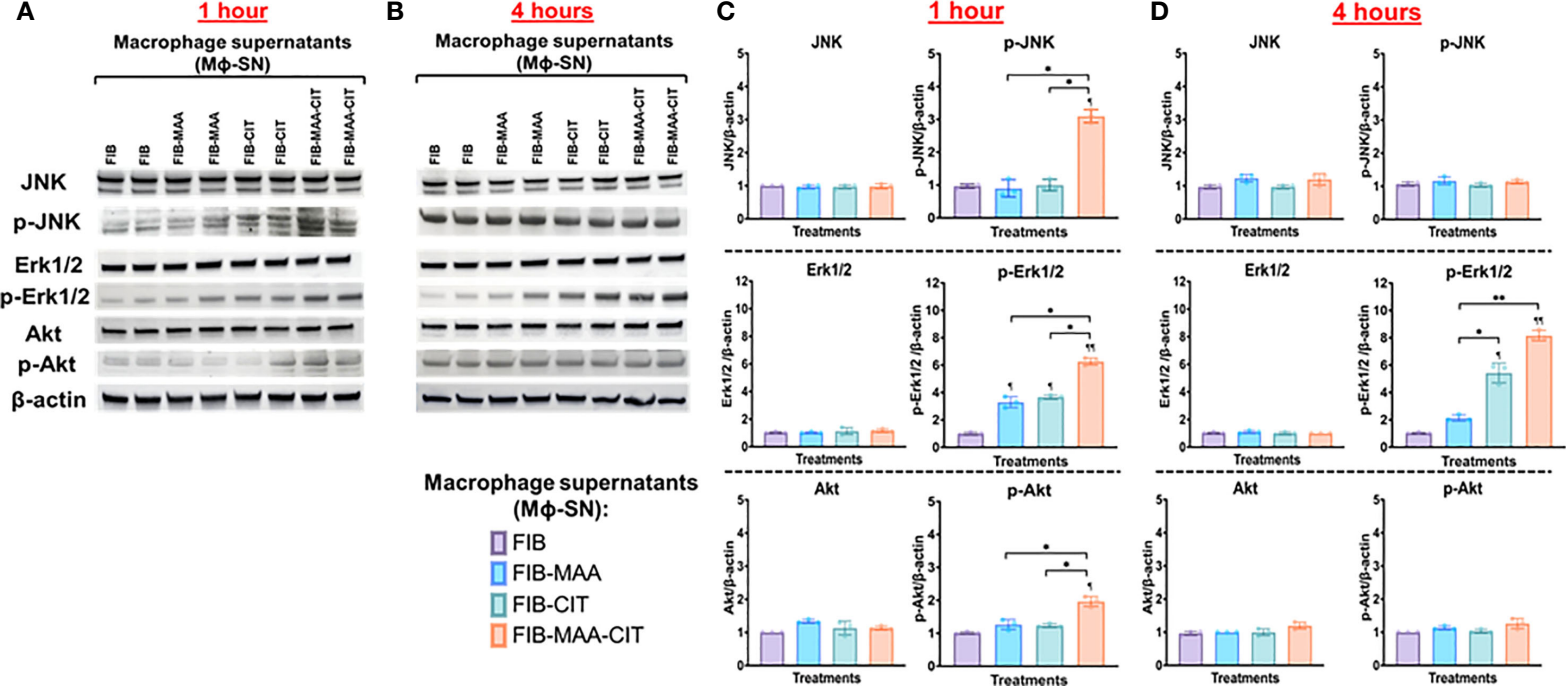
Western Blot of signaling pathways in stimulated HFLS-RA cells. HFLS-RA cells were treated with macrophage supernatants (Mϕ-SN) or direct antigen stimulation for 1 and 4 hours. Mϕ-SN were collected post-treatment of PMA-activated U-937 cells with the modified fibrinogen (FIB) antigens. Direct stimulation corresponds to treatment of HFLS-RA cells with the modified FIB antigens. HFLS-RA lysates probed with signaling molecule antibodies for **(A)** 1-hour and **(B)** 4-hour incubation. Densitometry of normalized values to β-actin of signaling molecules for **(C)** 1-hour and **(D)** 4-hour incubation. The data is represented as a mean of densitometry normalized values with SEM. Kruskal-Wallis non-parametric test comparing to Mϕ-SN^FIB^ or FIB are illustrated above each bar: ^¶¶^p<0.01, ^¶^p<0.05; n=3. Dunn’s multiple comparison test was used for comparisons made between groups are illustrated above each bar: **p<0.01, *p<0.05; n=3. Akt, Rho-family-alpha serine/threonine-protein kinase; FIB, native fibrinogen; FIB-MAA, MAA-modified fibrinogen; FIB-CIT, citrullinated fibrinogen; FIB-MAA-CIT, MAA and citrulline modified fibrinogen; JNK, Jun N-terminal kinase; Erk1/2, mitogen-activated protein kinase; Mϕ-SN; macrophage supernatants; p-Akt, phospho-Akt (Ser473); p-JNK, phospho-JNK (Thr183/Tyr185); p-Erk1/2, phospho-Erk1/2 (Thr202/Tyr204).

To delineate the signaling mechanisms involved, c-Jun N-terminal protein kinase (JNK), p38 mitogen activated protein kinase (MAPK), p44/42 MAPK (Erk1/2), Rho-family-alpha serine/threonine-protein kinase (Akt), paxillin, and focal adhesion kinase (FAK) were investigated in HFLS-RA cells incubated with Mϕ-SN. These signaling pathways were investigated based on their broad regulation of an aggressive phenotype in HFLS-RA cells (16, 50, 59). Incubation of HFLS-RA cells at 1-h and 4-h were optimal for phosphorylation of signaling molecules, whereas with 15-min and 30-min incubation only p-Erk1/2 was detected, and the band intensities for p-Erk1/2 were lower than 1-h and 4-h incubation (data not shown). At the time points examined, phosphorylation of p38 MAPK, paxillin, and FAK was not detected in HFLS-RA cells (data not shown). At 1-h, stimulations of HFLS-RA cells with Mϕ-SN^FIB-MAA^ and Mϕ-SN^FIB-CIT^ yielded 4-fold magnitude increases in Erk1/2 phosphorylation (p-Erk1/2) (vs. Mϕ-SN^FIB^), an increase not observed with JNK or Akt activation (**Figures 9A, C**). Exposure of HFLS-RA cells to Mϕ-SN^FIB-MAA-CIT^ increased phosphorylation (p-) of JNK (3-fold, p<0.05 vs. Mϕ-SN^FIB^), Erk1/2 (6-fold, p<0.01), and Akt (2-fold, p<0.05) at 1-h. Similar magnitudes of fold increase in phosphorylation were observed in the positive control (treatment of HFLS-RA with LPS; data not shown). After incubation for 4-h, only Erk1/2 remained phosphorylated (p-Erk1/2) following stimulation of HFLS-RA with Mϕ-SN^FIB-CIT^ (6-fold, p<0.05) and Mϕ-SN^FIB-MAA-CIT^ (8-fold, p<0.01) (**Figures 9B, D**). Protein expression of JNK, Erk1/2, and Akt were similar across the treatment groups at 1- and 4-h period (**Figure 9**). Notably, direct stimulation of HFLS-RA cells with the same antigens did not lead to phosphorylation of JNK, Erk1/2, and Akt at the time points examined above (data not shown). These experiments demonstrated that when exposed to Mϕ-SN, HFLS-RA cells activate signaling primarily through Erk1/2, JNK, and Akt pathways.

### 3.4 PDGF-BB release by macrophages

**Figure 10:**
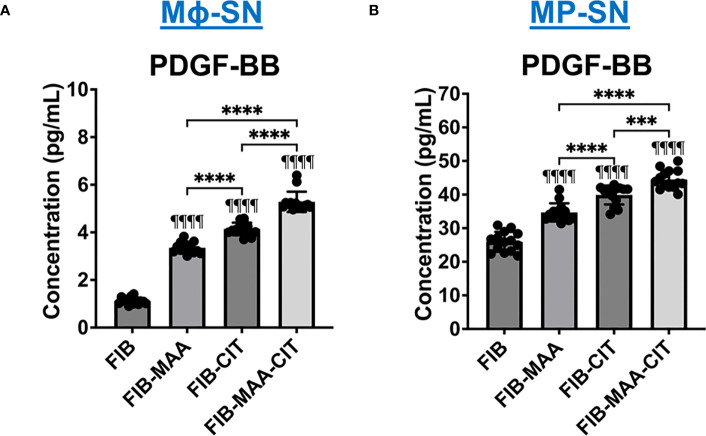
PDGF-BB concentration in supernatants from stimulated macrophages. **(A)** U-937 derived macrophage supernatants (Mϕ-SN) were collected post treatment with the modified fibrinogen (FIB) antigens. **(B)** PBMC derived macrophage supernatants (MP-SN) were collected post treatment with the modified FIB antigens. The table below the graph demonstrates mean of PDGF-BB concentration in pg/mL with SEM. Comparisons made to FIB are illustrated above each bar: ^¶¶¶¶^p<0.0001, n=14. One-way ANOVA with Tukey’s *post-hoc* test comparisons are illustrated between treatment groups, and only significant differences are illustrated: ****p<0.0001. FIB, native fibrinogen; FIB-MAA, MAA-modified fibrinogen; FIB-CIT, citrullinated fibrinogen; FIB-MAA-CIT, MAA and citrulline modified fibrinogen; PDGF-BB, platelet-derived growth factor-BB.

Due to the phosphorylation of JNK, Erk1/2, and Akt signaling molecules [pathways involved in PDGF-BB signaling (16, 50)] and transformation to an aggressive phenotype [PDGF-BB playing an established role in promoting this phenotype (60, 64)] in HFLS-RA cells stimulated with Mϕ-SN, we quantified PDGF-BB in Mϕ-SN (U-937 derived SN). Compared to stimulations with Mϕ-SN^FIB^, PDGF-BB was increased in Mϕ-SN following Mϕ stimulation with both FIB-CIT (p<0.0001) and FIB-MAA (p<0.0001). The highest concentration of PDGF-BB was observed after stimulations with FIB-MAA-CIT (p<0.0001 vs. all other groups) (**Figure 10A** **)**. A similar observation was made in the MP-SN derived from stimulated PBMCs (**Figure 10B**).

### 3.5 PDGF-BB activation of HFLS-RA cells

**Figure 11:**
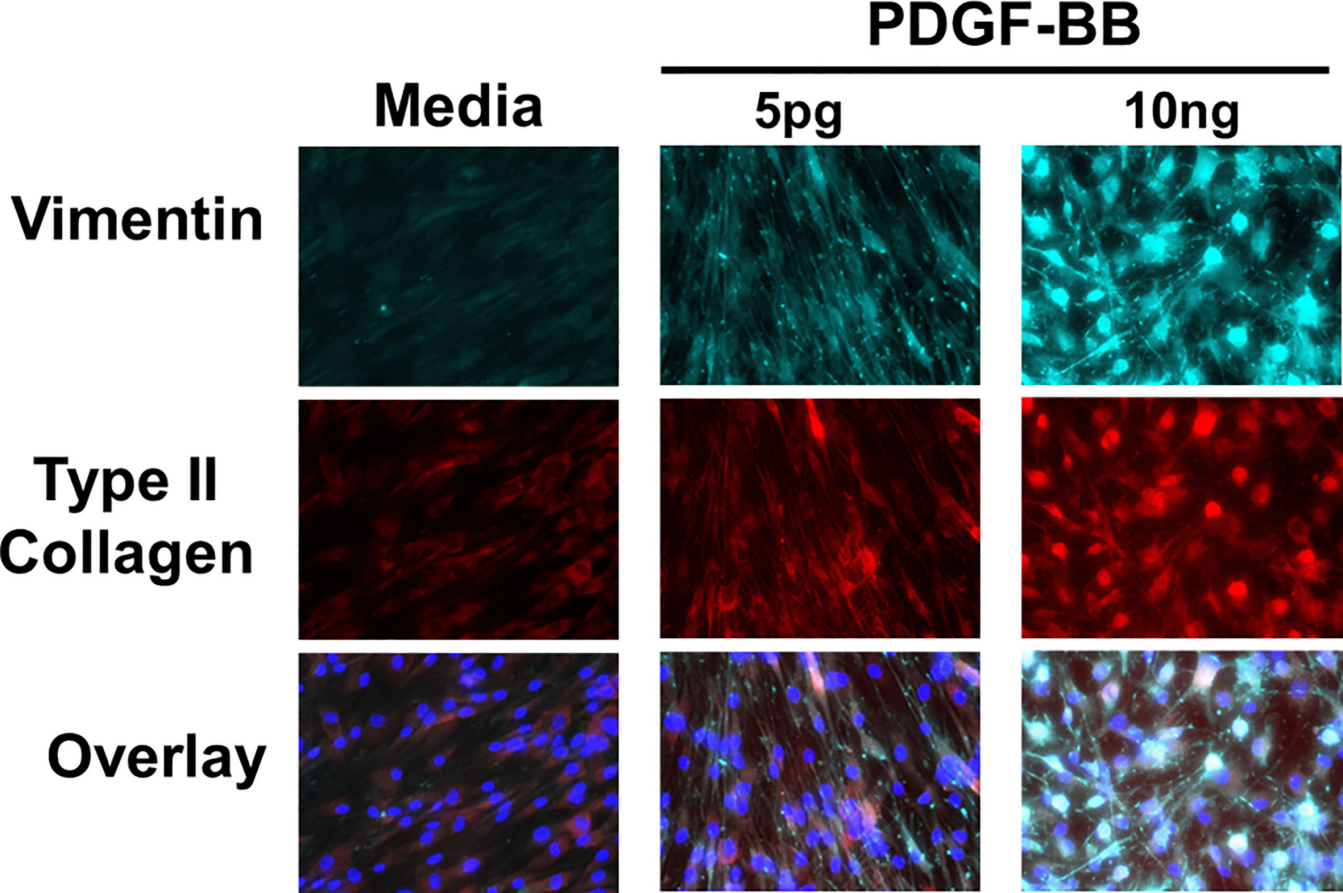
Fluorescent immunohistochemistry (IHC) of extracellular matrix (ECM) proteins in stimulated HFLS-RA cells. HFLS-RA cells were treated with 10ng/mL and 5pg/mL of PDGF-BB or media. Fluorescent IHC images of HFLS-RA cells stained for vimentin and type II collagen. N=6. PDGF-BB, platelet-derived growth factor-BB.

**Figure 12:**
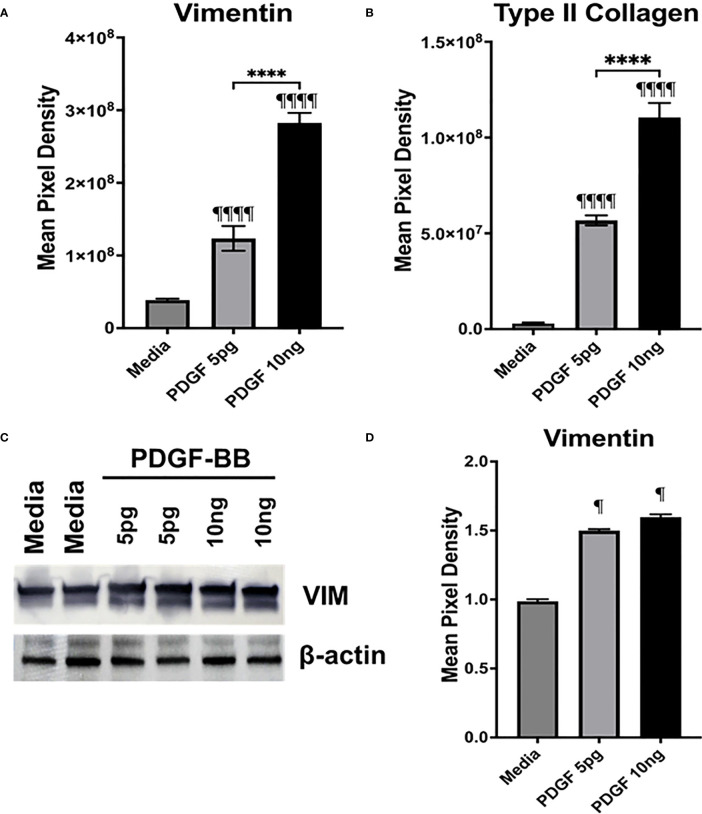
Quantification of fluorescent IHC and Western Blot of ECM proteins in stimulated HFLS-RA cells. HFLS-RA cells were treated with 10ng/mL and 5pg/mL of PDGF-BB or media. Quantification of fluorescent IHC images measuring mean pixel density for vimentin **(A)** and type II collagen **(B)**. **(C)** HFLS-RA lysates probed with anti-vimentin and anti-β-actin antibodies. **(D)** Densitometry of normalized values to β-actin of vimentin. The data is represented as a mean of meal pixel density (MPD) with SEM for fluorescent IHC and as a mean of densitometry normalized values with SEM for Western Blot. For IHC, comparisons made to media are illustrated above each bar: ^¶¶¶¶^p<0.0001, ^¶^p<0.05; n=6. One-way ANOVA with Tukey’s *post-hoc* test comparisons are illustrated between treatment groups, and only significant differences are illustrated: ****p<0.0001. For Western Blot, Kruskal-Wallis non-parametric test with *post-hoc* Dunn’s multiple comparison test to media are illustrated above each bar: ^¶^p<0.05; n=3. PDGF-BB, platelet-derived growth factor-BB.

**Figure 13:**
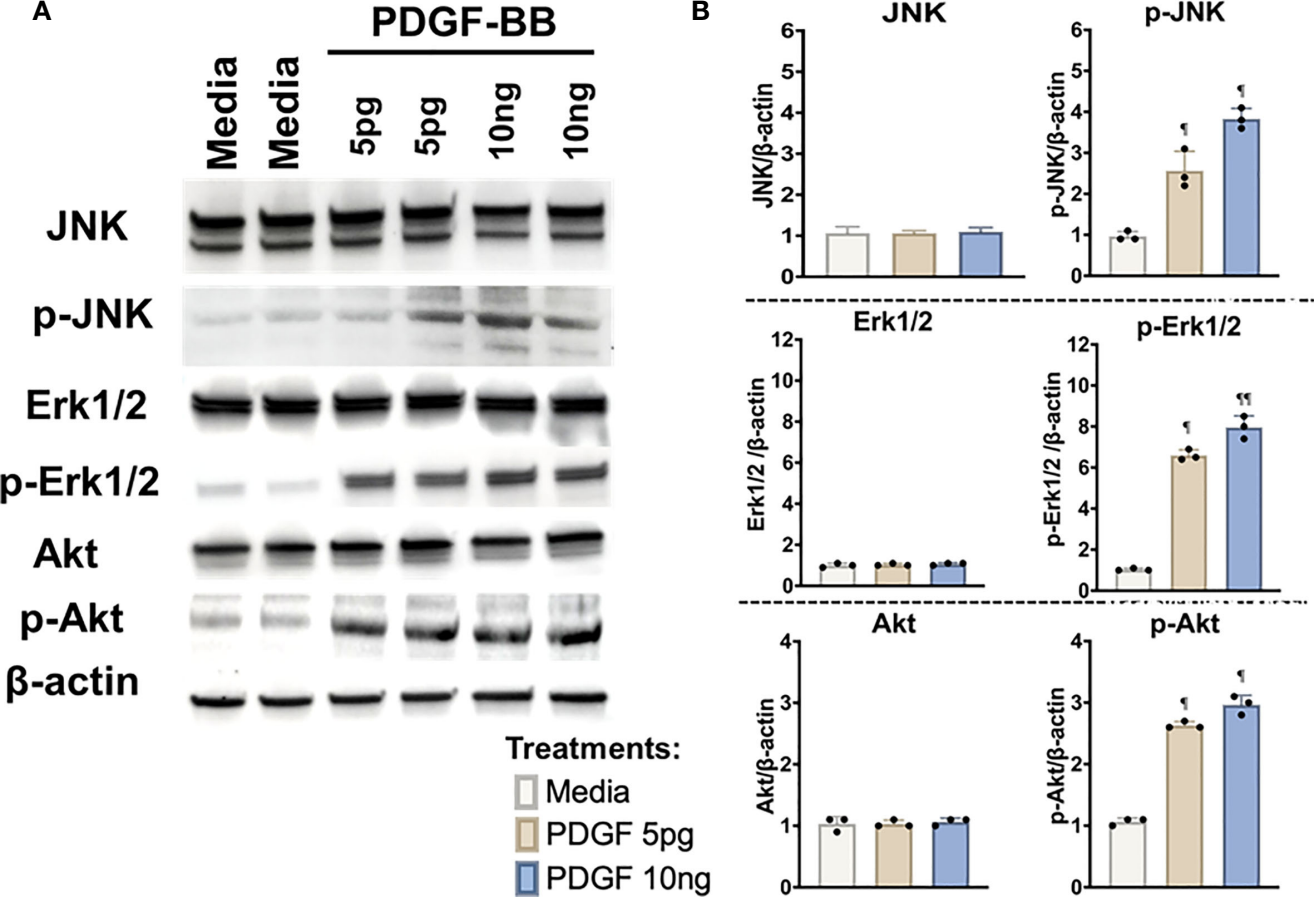
Western Blot of signaling pathways in stimulated HFLS-RA cells. HFLS-RA cells were treated with 10ng/mL and 5pg/mL of PDGF-BB or media for 1-hour. **(A)** HFLS-RA lysates probed with signaling molecule antibodies. **(B)** Densitometry of normalized values to β-actin of signaling molecules. The data is represented as a mean of densitometry normalized values with SEM. Kruskal-Wallis non-parametric test with *post-hoc* Dunn’s multiple comparison test to media are illustrated above each bar: ^¶^p<0.05, ^¶¶^p<0.01; n=3. Akt, Rho-family-alpha serine/threonine-protein kinase; JNK, Jun N-terminal kinase; Erk1/2, mitogen-activated protein kinase; p-Akt, phospho-Akt (Ser473); p-JNK, phospho-JNK (Thr183/Tyr185); p-Erk1/2, phospho-Erk1/2 (Thr202/Tyr204); PDGF-BB, platelet-derived growth factor-BB.

The presence of PDGF-BB in the Mϕ-SN prompted us to investigate the effect of PDGF-BB on HFLS-RA cellular morphology, ECM deposition, and signaling pathway activation. HFLS-RA cells were stimulated with 10ng and 5pg of PDGF-BB based on previous literature (60, 66) and mean PDGF-BB levels detected in Mϕ-SN, as described above. By fluorescent IHC, HFLS-RA cellular morphology appeared “spindle-shaped” only at 10ng PDGF-BB treatment (**Figure 11**). Additionally, vimentin and type II collagen deposition were significantly upregulated with 10ng PDGF-BB stimulation and to a lesser extent with 5pg in comparison to treatment with media (**Figures 12A, B**). By Western Blot, vimentin expression was significantly upregulated in PDGF-BB treated groups (p<0.05 vs. media; **Figures 12C, D** **)**. For signaling pathways, HFLS-RA cells increased expression of p-JNK, p-Erk1/2, and p-Akt at both 10ng and 5pg PDGF-BB treatment concentrations (p<0.05) compared to media at 1-h incubation period (**Figure 13**). The levels of JNK, Erk1/2, and Akt remained the same among the treatment groups. These results show that PDGF-BB is present in Mϕ-SN following stimulation with modified FIB and activates HLFS promoting an aggressive phenotype.

## 4 Discussion

To our knowledge, this report is the first to establish a cellular mechanism linking activated macrophages and synovial fibroblasts *via* responses to MAA-modified and citrullinated fibrinogen. Exposure to macrophage supernatants increased the expression of markers consistent with an aggressive HFLS-RA phenotype, as compared to direct stimulation with the same forms of modified fibrinogen. Further, HFLS-RA cells increased invasiveness and exhibited striking changes in cellular morphology, appearing “spindle-cell” shaped, following incubation with supernatants from macrophages stimulated with dually-modified fibrinogen and to a lesser extent with citrullinated fibrinogen. The synergistic effects of citrulline and MAA-modified fibrinogen on macrophages that in turn activate multiple components of an aggressive HFLS-RA phenotype implicates a role of multiple post-translational modifications in fibroblast transformation characterizing the development and progression of RA. Additionally, such distinctive morphological features and the magnitude of change in pro-inflammatory, pro-fibrotic, pro-invasive, and pro-chondrogenic markers, including the expression and deposition of ECM proteins, were not observed in HFLS from controls or patients with OA. This observation strongly indicates the presence of a uniquely aggressive phenotype in HFLS cells from patients with RA, a finding that has been reported by others (16, 60, 67, 68). Further, an aggressive phenotype of HFLS-RA cells has been reported to contain a subpopulation of type II collagen expressing synovial fibroblasts (chondrogenic marker), which are likely contributing to cell migration and invasiveness (51, 52, 55).

Experiments evaluating the contributions of macrophage supernatants to HFLS-RA activation were performed investigating multiple signaling pathways. These studies revealed that HFLS signaling in response to macrophage supernatants primarily occurred through Erk1/2, JNK, and Akt pathways. In fibroblasts, PDGF binding to PDGF-receptor is known to activate Akt, JNK, and predominantly Erk1/2 MAPK (60, 63, 67, 69). This distinctive signaling pattern implicated PDGF as a potential activating molecule in the supernatants of the stimulated macrophages. Likewise, PDGF has been implicated in RA pathogenesis as serum and synovial fluid concentrations are higher in patients with RA than in controls (65, 70, 71).

In this study, we measured the PDGF-BB isoform due to its recognized role in HFLS-RA cell activation. Several *in vitro* studies have demonstrated that HFLS-RA stimulation with PDGF-BB promotes robust fibroblast activation and increased ECM remodeling (60, 64, 66, 67, 72). With respect to signaling mechanisms in fibroblasts, PDGF-BB is known to activate Ras-Raf1-Mek1/2-Erk1/2, JNK, and Akt signaling pathways via increased phosphorylation (60–63). Erk1/2 and JNK are members of mitogen activated protein kinase family (MAPK). These pathways play a critical role in the activation of transcription factors for MMP gene expression in fibroblasts and contribute to tissue remodeling as well as fibroblast proliferation, migration, and invasiveness (73–75). Activation of the Akt signaling cascade contributes to several aspects of fibroblast activation with the most prominent roles being involvement in fibroblast proliferation and escape from apoptosis (60, 76). Beyond stimulated HFLS demonstrating characteristic involvement of the Erk1/2, JNK, and Akt pathways, other experiments were completed to further confirm PDGF-BB as a mediating soluble constituent in macrophage supernatants. Direct stimulation of HFLS with PDGF-BB yielded morphological changes and ECM deposition that paralleled those resulting from co-incubation with macrophage supernatants, particularly those generated after exposure to dually-modified fibrinogen.

Mean PDGF-BB concentrations in macrophage supernatants following stimulation with dually-modified fibrinogen were 5pg/mL, higher than that seen from macrophages stimulated with fibrinogen harboring only a single modification. Upon treatment of HFLS-RA with 5pg/mL of PDGF-BB, cells increased the expression of ECM proteins and upregulated phosphorylation of the same signaling pathways as treatment with macrophage supernatants. However, the same concentration PDGF-BB did not replicate the striking morphological changes that were observed following stimulation with supernatants from macrophages stimulated with dually-modified antigen. These morphologic changes were replicated in HFLS-RA cells only after exposure to PDGF-BB at concentrations of 10 ng/ml. While these findings implicate PDGF-BB as a mediating factor, it also strongly suggests that there are other soluble mediators in macrophage supernatants such as residual modified fibrinogen that impact the activation of HFLS-RA cells or that could act to lower the threshold of the necessary concentration of PDGF-BB to induce these changes in HFLS-RA cells.

It has been proposed that the combination of somatic mutations in key genes and the synovial inflammatory milieu promote HFLS-RA to display a unique phenotype (16, 17, 50, 77). HFLS-RA cells exhibit certain features, similar to transformed cells that possess elongated and oval morphology, demonstrate aggressive and hyperplastic properties, and increase ECM remodeling. Prior research has implicated a number of factors produced by immune cells that are present in RA synovium that might contribute to the transformation of HFLS-RA cells into a ‘aggressive’ phenotype (16, 17, 78–81). These include transforming growth factor-β (TGF-β), tumor necrosis factor-α (TNF-α), interleukin-1β (IL-1β), PDGF, and others. In idiopathic pulmonary fibrosis, a disease that has striking overlap with RA-associated interstitial lung disease in a usual interstitial pneumonia pattern, PDGF secretion from alveolar macrophages has been shown to promote fibroblast growth and proliferation (82). Our findings, in line with other studies (60, 64, 67, 80, 82), strongly implicate PDGF as one of the potential contributors to the pathophysiological changes in HFLS-RA.

The precise origin and timeline of transformation for HFLS-RA cells is currently unknown. However, HFLS-RA cells are among the primary drivers of synovitis in RA, contributing to both pannus formation and joint destruction (16, 18). Recent evidence suggests that clinically undetectable joint inflammation is present before the onset of classifiable RA (8), and infiltration of the joint by macrophages has been reported in the clinically uninvolved joints of RA patients and thus suggested to be an early harbinger of disease (83). An early role for macrophages and macrophage-fibroblast crosstalk in RA development is also supported by results of a separate study identifying serum PDGF concentration as the strongest biomolecular discriminator between individuals with pre-clinical disease and patients with recent-onset RA (70). Coupled with results herein, these data support the notion that modified proteins leading to macrophage activation and macrophage-fibroblast crosstalk play an important role in this transition.

Based on the findings in this study, we postulate that macrophages release PDGF in response to MAA- and citrulline-modified fibrinogen, which in turn activates HFLS-RA cells to express inflammatory, fibrotic, invasive, and chondrogenic markers that serve as cardinal features of the transformed RA synoviocyte. In pre-RA stages of synovitis, we speculate that the expression of the transformed synoviocyte is likely regulated by PDGF (and possibly other soluble mediators) in a paracrine fashion, released from macrophages activated by endogenous post-translationally modified proteins. Further, these effects were amplified with both citrulline and MAA modification of fibrinogen, providing a novel insight into the dual effects of multiple post-translational modifications in pre-RA stages of synovitis. Additional studies examining the role of PDGF receptor inhibition in attenuating biological responses from activated HFLS-RA cells are warranted.

We focused our studies on the impact of modified fibrinogen on macrophage and fibroblast activation. In the RA synovium, ACPA also target other post-translationally modified ECM proteins, such as vimentin, type II collagen, and many others (33, 34). MAA and/or citrulline modifications of vimentin or collagen may lead to different mechanistic responses due to variable immunogenicity. Similarly, we have evaluated responses in U-937 derived and PBMC derived macrophages, recognizing that synovial macrophages potentially exhibit different responses due to variable tissue-specificity. Additionally, due to the presence of autoantibodies prior to synovitis onset, future studies will be needed to examine the effect of ACPA and anti-MAA antibody immune complexes on cellular crosstalk that occurs between macrophages and HFLS-RA cells. In RT-PCR experiments, we did not examine some of the markers (IL-1β, IL-6, MMP-9, MMP-10, MMP-12, TGF-β) beyond gene expression due to a potential interference/confounding of these markers with the factors already present in macrophage supernatants. Additionally, other fibrosis-associated collagens were not evaluated in this study, instead we evaluated multiple components of aggressive phenotype in HFLS-RA cells, including pro-chondrogenic marker (type II collagen). Inhibition or silencing studies were not performed in this study and will be examined in future research, likely using small interfering RNA inhibitors and/or commercially available small molecule inhibitors of the PDGF-receptor (i.e., imatinib, nintedanib, or sunitinib; non-selective agents that also block tyrosine-kinase receptors). Instead, for these studies, we chose to evaluate the direct effects of PDGF on HFLS-RA cells, demonstrating similar responses to macrophage supernatants.

**Figure 14:**
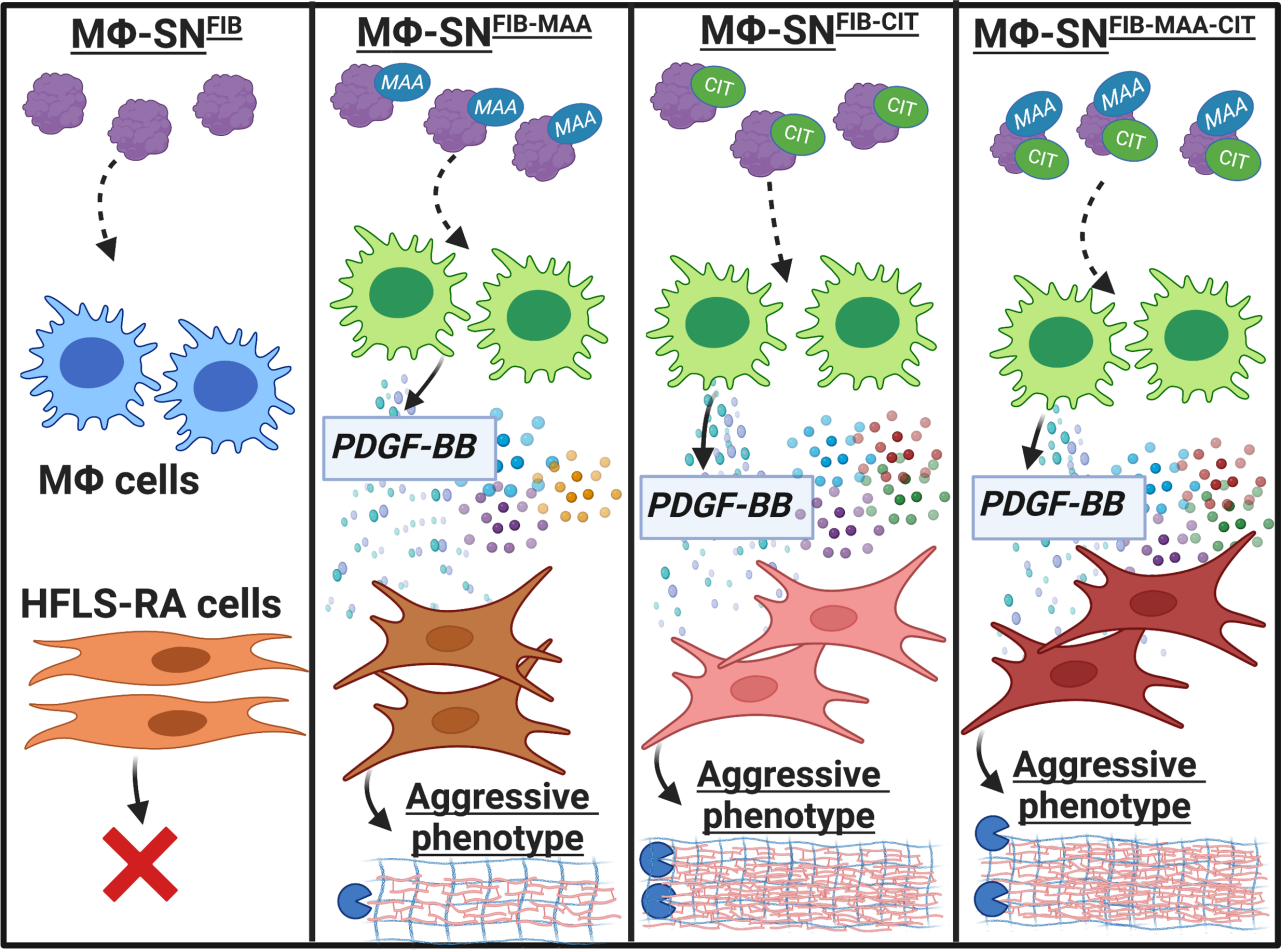
Summary of our findings. Our data provides initial evidence that PDGF-BB, present in Mϕ-SN in response to stimulation with modified fibrinogen (FIB), contributes to the phenotypic transformation of HFLS-RA cells, promoting an aggressive phenotype. Macrophages stimulated with FIB modified with both citrulline and malondialdehyde-acetaldehyde (MAA) (vs. stimulation with unmodified FIB) lead to the generation of supernatants that are characterized by increased PDGF-BB concentrations and promote the transformation of HFLS-RA cells into an aggressive phenotype. The figure was created using BioRender. HFLS-RA, human fibroblast like synoviocytes from RA synovium; Mϕ, macrophages; Mϕ-SN, macrophage supernatants; PDGF-BB, platelet derived growth factor-BB. Created with BioRender.com.

A summary of our findings is illustrated in **Figure 14**. Our data provide initial evidence that PDGF serves as a soluble factor released from macrophages in response to endogenous modified proteins and contributes to the phenotypic transformation of HFLS-RA cells. The novel findings from these experiments specifically implicate macrophages activated with dually modified fibrinogen in promoting macrophage secretion of PDGF, which enhances activation of multiple components of an aggressive fibroblast phenotype. Further, macrophages stimulated with dually modified (MAA and citrulline) fibrinogen promoted stronger HFLS-RA responses than either single protein modification. These results further demonstrate that both MAA and citrulline modifications likely play a potentially synergistic role in driving macrophage-fibroblast crosstalk contributing to the initiation and propagation of synovial inflammation in RA.

## Data availability statement

The original contributions presented in the study are included in the article/**Supplementary Material**. Further inquiries can be directed to the corresponding author.

## Ethics statement

Ethical approval was not required for the studies on humans in accordance with the local legislation and institutional requirements because only commercially available established cell lines were used.

## Author contributions

All authors were involved in drafting the article or revising it critically important for intellectual content, and all authors approved the final version to be published. NA had full access to all the data in the study and takes responsibility for the integrity of the data and accuracy of the data analysis. Study conception and design, NA, MD, BE, DR, GT, and TM. Acquisition of data, NA, MD, CH, JM, ER, ED, GT, and TM. Analysis and interpretation of data, NA, MD, BE, CH, JM, ER, ED, DR, GT, and TM. Original draft writing, NA. Review and editing of the draft, NA, MD, BE, CH, JM, ER, ED, DR, GT, and TM.
