# Supplementary material for: Citrullinated and malondialdehyde-acetaldehyde modified fibrinogen activates macrophages and promotes an aggressive synovial fibroblast phenotype in patients with rheumatoid arthritis
Source: Front Immunol. 2023 Aug 16;14:1203548. doi: 10.3389/fimmu.2023.1203548 (PMC10467288; doi:10.3389/fimmu.2023.1203548)
Supplement: Supplementary file 1 [file Image_1.pdf]

Supplemental Material:

HFLS-RA cells

Incubation time in hours (h)		Macrophage supernatants (Mϕ-SN):				Direct stimulation:			
		FIB	FIB-MAA	FIB-CIT	FIB-MAA-CIT	FIB	FIB-MAA	FIB-CIT	FIB-MAA-CIT
8-h	***Pro-inflammatory genes***								
	IL-1β	1.0 (0.0)	4.0 (0.2)	**6.2 (0.2)***	**6.7 (0.3)***	1.1 (0.1)	0.7 (0.0)	**11.7 (1.0)***	**8.4 (0.8)***
	IL-6	1.0 (0.1)	2.3 (0.1)	**5.6 (0.2)***	**4.2 (0.2)***	1.0 (0.0)	0.6 (0.1)	**26.4 (1.8)***	**16.0 (1.5)***
	Pro-fibrotic genes								
	TGF-β	0.9 (0.1)	1.0 (0.2)	1.1 (0.1)	1.0 (0.1)	1.0 (0.1)	0.8 (0.2)	1.0 (0.1)	1.1 (0.2)
	VIM	0.9 (0.2)	1.1 (0.1)	1.2 (0.1)	0.9 (0.1)	1.0 (0.1)	1.0 (0.1)	1.0 (0.2)	0.9 (0.2)
	Pro-invasiveness genes								
	MMP-9	1.0 (0.2)	**3.3 (0.2)***	2.5 (0.1)	**3.4 (0.2)***	1.0 (0.2)	1.2 (0.2)	**2.1 (0.1)***	1.7 (0.1)
	MMP-10	1.0 (0.1)	1.9 (0.1)	2.9 (0.0)	**5.1 (0.2)***	1.1 (0.1)	1.3 (0.1)	**2.7 (0.3)***	2.2 (0.1)
	MMP-12	1.0 (0.0)	2.2 (0.1)	**7.3 (0.1)***	**9.7 (0.6)***	1.1 (0.2)	1.3 (0.1)	**6.8 (0.2)***	**6.7 (0.3)***
	Pro-chondrogenesis genes								
	COL2A1	1.0 (0.1)	1.4 (0.2)	**2.4 (0.2)***	0.4 (0.1)	1.0 (0.0)	0.5 (0.0)	**2.1 (0.2)***	0.5 (0.1)
24-h	***Pro-inflammatory genes***								
	IL-1β	1.0 (0.1)	1.6 (0.2)	**3.0 (0.1)***	2.4 (0.2)	0.9 (0.2)	0.6 (0.0)	**6.5 (0.5)***	2.1 (0.3)
	IL-6	1.0 (0.0)	1.5 (0.0)	**2.6 (0.2)***	**2.6 (0.2)***	1.0 (0.0)	0.8 (0.2)	**8.9 (0.9)***	2.9 (0.2)
	Pro-fibrotic genes								
	TGF-β	1.0 (0.1)	1.3 (0.2)	**2.4 (0.4)**$^{\#}$	**4.9 (0.6)**$^{*¥}$	1.0 (0.2)	0.9 (0.1)	1.0 (0.3)	1.3 (0.2)
	VIM	0.9 (0.1)	0.7 (0.1)	**1.9 (0.1)***	**2.8 (0.3)**$^{*¥}$	1.0 (0.1)	1.0 (0.0)	0.8 (0.2)	1.9 (0.1)
	Pro-invasiveness genes								
	MMP-9	1.1 (0.1)	0.8 (0.2)	**23.1 (2.5)***	**35.2 (2.9)***	0.9 (0.1)	1.0 (0.1)	0.9 (0.3)	0.9 (0.1)
	**MMP-10**	0.9 (0.1)	1.0 (0.1)	**3.6 (0.3)***	**4.3 (0.9)***	1.1 (0.1)	0.9 (0.2)	0.8 (0.1)	1.0 (0.1)
	**MMP-12**	1.1 (0.1)	1.6 (0.1)	**11.5 (1.7)***	**19.0 (5.3)***	1.1 (0.1)	1.0 (0.2)	2.3 (0.3)	1.0 (0.2)
	Pro-chondrogenesis genes								
	COL2A1	1.0 (0.1)	1.0 (0.4)	**3.9 (0.4)***	**5.4 (0.9)***	0.9 (0.1)	1.1 (0.1)	1.3 (0.4)	0.3 (0.0)

Supplemental Figure 1. Heatmap of RT-PCR for aggressive phenotype genes in stimulated HFLS-RA cells. HFLS-RA cells were treated with macrophage supernatants (Mϕ-SN) or direct antigen stimulation for 8- and 24- hours. Mϕ-SN were collected post-treatment of PMA-activated U-937 cells with the modified fibrinogen (FIB) antigens. Direct stimulation corresponds to treatment of HFLS cells with unmodified and modified FIB. Pro-inflammatory genes: *IL-1β, IL-6*. Pro-fibrotic genes: *TGF-β, VIM.* Pro-invasiveness genes*: MMP-9, MMP-10, MMP-12. Pro-chondrogenesis gene: COL2A1*. Data are shown as a mean of relative quantity (Rq) with standard error of the mean (SEM) of markers. Red boxes represent increased mRNA levels, while green boxes represent decreased mRNA levels. Gradient transition shows lowest to highest expression by color compared to FIB or Mϕ-SN^{FIB} (Rq=1.0) represented as white color. Kruskal-Wallis non-parametric test with post-hoc Dunn's multiple comparison test are shown when treatment groups were compared to Mϕ-SN^{FIB} or FIB, *p<0.05, n=3.
Abbreviations: COL2A1, pro-alpha1 chain of type II collagen, FIB, native fibrinogen; FIB-MAA, MAA-modified fibrinogen; FIB-CIT, citrullinated fibrinogen; FIB-MAA-CIT, MAA and citrulline modified fibrinogen; HFLS, human fibroblast-like synoviocytes; IL-interleukin; MMP, metalloproteinase; Mϕ-SN; macrophage supernatants; TGF-β, transforming growth factor beta; VIM, vimentin.

HFLS-OA cells

Incubation time in hours (h)		Macrophage supernatants (Mϕ-SN):				Direct stimulation:			
		FIB	FIB-MAA	FIB-CIT	FIB-MAA-CIT	FIB	FIB-MAA	FIB-CIT	FIB-MAA-CIT
	Pro-inflammatory genes								
8-h	***IL-1β***	1.0 (0.2)	1.4 (0.2)	**3.3 (0.1)***	**3.2 (0.3)***	1.0 (0.0)	1.1 (0.1)	**7.3 (0.6)***	3.8 (0.2)
	IL-6	1.0 (0.1)	1.4 (0.2)	**4.8 (0.5)***	2.8 (0.2)	1.0 (0.2)	0.8 (0.1)	**7.5 (0.5)***	3.9 (0.4)
	Pro-fibrotic genes								
	TGF-β	1.1 (0.1)	0.8 (0.2)	1.1 (0.1)	1.1 (0.1)	1.0 (0.1)	1.1 (0.1)	1.0 (0.2)	1.1 (0.2)
	VIM	1.0 (0.1)	0.8 (0.1)	1.3 (0.1)	0.8 (0.2)	1.0 (0.1)	1.0 (0.0)	1.1 (0.2)	1.0 (0.3)
	Pro-invasiveness genes								
	MMP-9	1.0 (0.2)	**3.3 (0.2)***	2.5 (0.1)	**3.4 (0.2)***	1.0 (0.2)	1.2 (0.2)	**2.1 (0.1)***	1.7 (0.1)
	MMP-10	1.0 (0.1)	1.9 (0.1)	2.9 (0.0)	**5.1 (0.2)***	1.1 (0.1)	1.3 (0.1)	**2.7 (0.3)***	2.2 (0.1)
	MMP-12	1.0 (0.0)	2.2 (0.1)	**7.3 (0.1)***	**9.7 (0.6)***	1.1 (0.2)	1.3 (0.1)	**6.8 (0.2)***	**6.7 (0.3)***
	Pro-chondrogenesis genes								
	COL2A1	1.0 (0.2)	1.2 (0.1)	**2.2 (0.1)***	0.8 (0.2)	1.0 (0.0)	1.2 (0.2)	**1.7 (0.1)***	1.2 (0.1)

Incubation time in hours (h)		FIB	FIB-MAA	FIB-CIT	FIB-MAA-CIT	FIB	FIB-MAA	FIB-CIT	FIB-MAA-CIT
	Pro-inflammatory genes								
24-h	***IL-1β***	1.0 (0.1)	1.2 (0.1)	**2.1 (0.1)***	1.9 (0.0)	1.1 (0.1)	1.3 (0.2)	**3.8 (0.6)***	2.5 (0.2)
	IL-6	1.1 (0.1)	1.2 (0.1)	**1.9 (0.1)***	1.5 (0.1)	1.0 (0.1)	1.2 (0.1)	**2.7 (0.1)***	**2.1 (0.1)***
	Pro-fibrotic genes								
	TGF-β	1.0 (0.1)	1.2 (0.1)	**1.9 (0.0)***	1.3 (0.2)	1.0 (0.0)	1.4 (0.2)	1.6 (0.1)	0.8 (0.2)
	VIM	1.0 (0.2)	2.2 (0.1)	2.8 (0.1)	**4.2 (0.3)***	1.0 (0.1)	1.3 (0.0)	1.1 (0.1)	0.7 (0.0)
	Pro-invasiveness genes								
	MMP9	1.0 (0.1)	1.1 (0.1)	3.8 (0.3)	**3.5 (0.3)***	1.0 (0.1)	1.4 (0.1)	1.8 (0.0)	**2.3 (0.4)***
	**MMP10**	1.0 (0.0)	1.6 (0.1)	2.7 (0.1)	**3.0 (0.3)***	1.0 (0.1)	0.9 (0.2)	1.3 (0.1)	0.7 (0.2)
	**MMP12**	1.0 (0.1)	2.0 (0.2)	3.1 (0.2)	**5.4 (0.6)***	1.0 (0.1)	1.1 (0.1)	1.0 (0.1)	1.2 (0.2)
	Pro-chondrogenesis genes								
	COL2A1	1.0 (0.1)	2.7 (0.3)	2.8 (0.2)	**4.3 (0.5)***	1.0 (0.1)	**1.8 (0.1)***	1.4 (0.3)	1.5 (0.3)

Supplemental Figure 2. Heatmap of RT-PCR for pro-inflammatory and pro-fibrotic genes in stimulated HFLS-OA cells. HFLS-OA cells were treated with macrophage supernatants (Mϕ-SN) or direct antigen stimulation for 8- and 24- hours. Mϕ-SN were collected post-treatment of PMA-activated U-937 cells with the modified fibrinogen (FIB) antigens. Direct stimulation corresponds to treatment of HFLS cells with unmodified and modified FIB. Pro-inflammatory genes: *IL-1β, IL-6*. Pro-fibrotic genes: *TGF-β, VIM.* Pro-invasiveness genes*: MMP-9, MMP-10, MMP-12. Pro-chondrogenesis gene: COL2A1*. Data are shown as a mean of relative quantity (Rq) with standard error of the mean (SEM) of markers. Red boxes represent increased mRNA levels, while green boxes represent decreased mRNA levels compared to Mϕ-SN^{FIB} or FIB. Gradient transition shows lowest to highest expression by color with baseline (Rq=1.0) represented as white color. Kruskal-Wallis non-parametric test with post-hoc Dunn's multiple comparison test are shown when treatment groups were compared to Mϕ-SN^{FIB} or FIB, *$p<0.05$, n=3.

Abbreviations: COL2A1, pro-alpha1 chain of type II collagen, FIB, native fibrinogen; FIB-MAA, MAA-modified fibrinogen; FIB-CIT, citrullinated fibrinogen; FIB-MAA-CIT, MAA and citrulline modified fibrinogen; HFLS, human fibroblast-like synoviocytes; IL-interleukin; MMP, metalloproteinase; Mϕ-SN; macrophage supernatants; TGF-β, transforming growth factor beta; VIM, vimentin.

A) HFLS-RA stimulated with MP-SN

	Macrophage supernatants (MP-SN):			
	FIB	FIB-MAA	FIB-CIT	FIB-MAA-CIT
IL-6	1.0 (0.1)	1.6 (0.2)	19.7 (3.8)	**43.1 (20.9)***
MMP-9	1.0 (0.2)	1.7 (0.2)	4.1 (1.0)	**6.1 (2.4)***
MMP-10	1.0 (0.2)	1.9 (0.3)	3.8 (1.3)	**7.5 (2.3)***

B) HFLS control stimulated with Mϕ-SN

	Macrophage supernatants (Mϕ-SN):			
	FIB	FIB-MAA	FIB-CIT	FIB-MAA-CIT
IL-6	1.0 (0.2)	0.9 (0.1)	**2.4 (0.3)***	1.8 (0.1)
MMP-9	1.0 (0.2)	1.7 (0.0)	2.2 (0.1)	**2.7 (0.1)***
MMP-10	1.0 (0.1)	1.4 (0.1)	**2.5 (0.2)***	**2.5 (0.3)***

Supplemental Figure 3. Heatmaps of RT-PCR for markers in stimulated HFLS cells. (A) HFLS-RA cells were treated with MP-SN for 8 hours. MP-SN corresponds to treatment of HFLS-RA cells with PBMC supernatants (MP-SN) from PBMC stimulation with the modified fibrinogen (FIB) antigens. **(B)** HFLS control cells were treated with Mϕ-SN for 8 hours. HFLS control cells were treated with Mϕ-SN collected post-treatment of U-937 cells with the modified FIB antigens. Red boxes represent increased mRNA levels, while green boxes represent decreased mRNA levels. Gradient transition shows lowest to highest expression by color with MP-SN^{FIB} or FIB (Rq=1.0) represented as white color. Kruskal-Wallis non-parametric test with post-hoc Dunn's multiple comparison test are shown when treatment groups were compared to Mϕ-SN^{FIB} or FIB, *p<0.05, n=3.

Abbreviations: FIB, native fibrinogen; FIB-MAA, MAA-modified fibrinogen; FIB-CIT, citrullinated fibrinogen; FIB-MAA-CIT, MAA and citrulline modified fibrinogen; IL-interleukin; MMP, metalloproteinase.
